# Supplementary material for: CASNET2: evaluation of an electronic safety netting cancer toolkit for the primary care electronic health record: protocol for a pragmatic stepped-wedge RCT
Source: BMJ Open. 2020 Aug 24;10(8):e038562. doi: 10.1136/bmjopen-2020-038562 (PMC7449309; doi:10.1136/bmjopen-2020-038562)
Supplement: Supplementary data [file bmjopen-2020-038562supp001.pdf]

Appendix A: List of study sites

The list below is of NHS Local Clinical Research Networks within whose areas the general practices in the study will be located. At the time of publication, identification and recruitment of individual general practices for the study had not been completed. A complete list of individual sites will be available on request from the study team upon completion of study recruitment.

- North East and North Cumbria
- North West Coast
- Yorkshire and Humber
- Greater Manchester
- West Midlands
- West of England
- Thames Valley and South Midlands
- Eastern
- Kent, Surrey and Sussex
- Wessex
- South West Peninsula
- South London
- North West London
