# Supplementary material for: CASNET2: evaluation of an electronic safety netting cancer toolkit for the primary care electronic health record: protocol for a pragmatic stepped-wedge RCT
Source: BMJ Open. 2020 Aug 24;10(8):e038562. doi: 10.1136/bmjopen-2020-038562 (PMC7449309; doi:10.1136/bmjopen-2020-038562)
Supplement: Supplementary data [file bmjopen-2020-038562supp002.pdf]

RISP
Research Information Sheet for Practices

**Study Title**	***CASNET2: Evaluation of an e-safety netting cancer template in primary care: a pragmatic stepped-wedge RCT***
**Principal Investigator(s) & Institution**	***Clare Bankhead, University of Oxford***
**NIHR Portfolio Ref**	**REC: TBC**<br>**IRAS ID: 269169**<br>**CPMS ID: TBC**
**Planned start date/ Duration of study recruitment at site**	Planned start date: 1 Sept 2019<br>12 months study duration – active period may be between 2 and 12 months depending on randomisation
**Study Funding Source**	Cancer Research UK
**Study Aim and Objectives**	The main aim of the study is to evaluate the E-safety netting (E-SN) toolkit, which is available as part of the EMIS electronic health record system. Our primary objective is to compare diagnostic outcomes for cancer (such as the time to diagnosis) during periods of time when the E-SN toolkit is turned off, compared with those during periods when the E-SN toolkit is turned on.<br>We also wish to compare consultation outcomes (such as the number of GP consultations before referral) during periods of time when the E-SN toolkit is turned off, compared with periods when the E-SN toolkit is turned on, to measure variation in the use of the E-SN toolkit between GP practices, and to describe the clinical situations for which the E-SN toolkit is used by GPs.<br>The E-SN toolkit is designed to assist with safety netting by providing a pro-active approach to tracking patients, ensuring that test requests, referrals and concerning symptoms are followed up in a timely manner.<br>We plan to recruit 60 general practices who are not currently using the E-SN toolkit, and randomise them in clusters (groups) of 10. Each cluster will have the E-SN toolkit turned on at a different time during the 12 months of the study. Once the E-SN toolkit is turned on, the GPs in the practice will be able to use it when treating any patient they think would benefit from it, although we expect that it will be of most useful when treating patients with symptoms that might indicate cancer.<br>We will collect data from the electronic patient record system from the 12 months of the study and the 24 months before the start of the study to understand whether the introduction of the E-SN toolkit makes any difference to the diagnosis of cancer, and particular to how quickly patients are diagnosed. We will only extract records from patients who are over 18, and who have not opted out of the research.

Fleming S, *et al. BMJ Open* 2020; 10:e038562. doi: 10.1136/bmjopen-2020-038562

**Site target**	Not applicable as intervention is at practice rather than patient level
**Staff involvement at Practice**	General practitioners<br>Administrator
**Total funding paid**	Up to £500<br>• £100 for activating the template and attending the toolkit training provided by the study team<br>• £250 – for using the template as required during the study period<br>• £150 – to cover administrative time for keeping record of weekly searches and sending monthly reports to the study team
**Eligibility Criteria**	**Inclusion criteria:**<br>• Practice is actively contributing data to RCGP RSP<br>• Utilises EMIS electronic health records system<br>• Data available for the previous 24 months<br>• (Patients) Male or female, aged 18 years or above<br>**Exclusion criteria:**<br>• Practices that express an interest, but are not fully set up to start downloading data.<br>• Any practice already deploying the E-Safety netting toolkit within EMIS
**STUDY ACTIVITIES:**	
**Site Activities**	• Attend training in use of E-safety netting toolkit<br>• Activate E-safety netting toolkit in EMIS on date instructed by study team<br>• GPs to use E-safety netting toolkit in routine care when judged appropriate<br>• Administrator to perform weekly search in EMIS for open diary entries and download these to an Excel spreadsheet<br>• Downloaded Excel spreadsheets of open diary entries to be provided on a monthly basis to study team.
**Patient Activities**	None
**Recruitment data process**	Recruitment will be reported by the study team, so no recruitment upload is required by practices.
**Study specific training**	• GPs at participating sites will receive training in use of the E-safety netting toolkit.<br>• The study team will also provide training and support for administrators to assist with weekly searching for open diary entries.
**Possible risks**	• Use of the E-safety netting toolkit may cause a slight increase in overall consultation length, although this is expected to be of the order of tens of seconds<br>• Reduced trust in the health system due to patients becoming aware that their data is being used for research without explicit consent
**GDPR and confidentiality information**	Responsible members of the University of Oxford may be given access to data for monitoring and/or audit of the study to ensure that the research is complying with applicable regulations.<br>Data protection regulation requires that we state the legal basis for processing information about your practice and the patients registered there. In the case of research, this is 'a task in the public interest.' The University of Oxford is the data controller and is responsible for looking after this information and using it properly.<br>We will be using information from medical records in order to undertake this study. Patients who have opted not to share their information for disease surveillance will be respected by RCGP RSC and by the research team. The research team will not extract records of patients who have registered opt-out codes in the GP information system.

Fleming S, et al. BMJ Open 2020; 10:e038562. doi: 10.1136/bmjopen-2020-038562

	Outcome data will be extracted from the EHR by the SQL developer and provided in a pseudonymised form to the analysis team.<br><br>You can find out more about how we use information from your practice and the patients registered there by contacting clare.bankhead@phc.ox.ac.uk or susannah.fleming@phc.ox.ac.uk
**Withdrawal from study**	Participation in the study is voluntary. If a practice chooses to withdraw from the study, data collected up to that point will be included in analyses. A practice may request upon withdrawal that data collected up to the point of withdrawal should not be used for analysis.<br>Practices who withdraw will receive the payment for activation and training, but payments for using the template and sending monthly reports of searches will be provided pro-rata for the period of time spent with the E-SN toolkit turned on before withdrawal.
**Making a complaint**	The University of Oxford, as Sponsor, has appropriate insurance in place in the unlikely event that you suffer any harm as a direct consequence of your participation in this study. NHS indemnity operates in respect of the clinical treatment which is provided.<br>If you wish to complain about any aspect of the way in which you have been approached or treated, or how your information is handled during the course of this study, you should contact Clare Bankhead on 01865 289351, or by email: clare.bankhead@phc.ox.ac.uk. Alternatively, you may contact the University of Oxford Clinical Trials and Research Governance (CTRG) office on 01865 616480, or the head of CTRG, email ctrg@admin.ox.ac.uk.
**Research Sponsor**	University of Oxford<br>ctrg@admin.ox.ac.uk
**General study contact**	Manasa Tripathy, Practice Liason Officer<br>manasa.tripathy@phc.ox.ac.uk

Fleming S, *et al. BMJ Open* 2020; 10:e038562. doi: 10.1136/bmjopen-2020-038562
