# Supplementary material for: CASNET2: evaluation of an electronic safety netting cancer toolkit for the primary care electronic health record: protocol for a pragmatic stepped-wedge RCT
Source: BMJ Open. 2020 Aug 24;10(8):e038562. doi: 10.1136/bmjopen-2020-038562 (PMC7449309; doi:10.1136/bmjopen-2020-038562)
Supplement: Supplementary data [file bmjopen-2020-038562supp003.pdf]

CASNET2 Study

RCGP
Research & Surveillance Centre Practice

This practice is taking part in the CASNET2 study. This is a study run by researchers at the University of Oxford looking at a new electronic safety netting tool which is part of the practice computer system. The tool helps GPs to make sure that patients get followed up if they have symptoms that might lead to a new diagnosis.

The researchers are hoping to find out whether the tool helps to reduce the time it takes for people to get a diagnosis. They will be using anonymised patient data from the practice computer system to find out how well the tool works. Using data in this way will not affect your privacy or care, and no-one will be able to identify you personally, but please speak to your GP if you have any concerns, or if you wish to opt out of the study.

For further information please visit http://www.rcgp.org.uk/rsc or contact:

Prof Simon de Lusignan
RSC Director
MedicalDirectorRSC@rcgp.org.uk

Dr Lucy Moore
Research Facilitator
practiceenquiries@phc.ox.ac.uk

Royal College of General Practitioners (RCGP) Research and Surveillance Centre (RSC)

21 Nov 2019 version 1.5
