# Supplementary material for: CASNET2: evaluation of an electronic safety netting cancer toolkit for the primary care electronic health record: protocol for a pragmatic stepped-wedge RCT
Source: BMJ Open. 2020 Aug 24;10(8):e038562. doi: 10.1136/bmjopen-2020-038562 (PMC7449309; doi:10.1136/bmjopen-2020-038562)
Supplement: Author's manuscript [file bmjopen-2020-038562.draft_revisions.pdf]

BMJ Open is committed to open peer review. As part of this commitment we make the peer review history of every article we publish publicly available.

When an article is published we post the peer reviewers' comments and the authors' responses online. We also post the versions of the paper that were used during peer review. These are the versions that the peer review comments apply to.

The versions of the paper that follow are the versions that were submitted during the peer review process. They are not the versions of record or the final published versions. They should not be cited or distributed as the published version of this manuscript.

BMJ Open is an open access journal and the full, final, typeset and author-corrected version of record of the manuscript is available on our site with no access controls, subscription charges or pay-per-view fees (http://bmjopen.bmj.com).

If you have any questions on BMJ Open's open peer review process please email info.bmjopen@bmj.com

BMJ Open

CASNET2: Evaluation of an Electronic Safety Netting (E-SN) cancer toolkit for the primary care electronic health record: protocol for a pragmatic stepped-wedge RCT

Journal:	*BMJ Open*
Manuscript ID	bmjopen-2020-038562
Article Type:	Protocol
Date Submitted by the Author:	15-Mar-2020
Complete List of Authors:	Fleming, Susannah; University of Oxford, Nuffield Department of Primary Care Health Sciences<br>Nicholson, Brian; University of Oxford, Nuffield Dept Primary Care Health Sciences<br>Bhuiya, Afsana; North Central and East London Cancer Alliance<br>de Lusignan, Simon; University of Oxford, Nuffield Department of Primary Care Health Sciences; University of Surrey, Department of Clinical and Experimental Medicine<br>Hirst, Yasemin; University College London, Epidemiology and Public Health<br>Hobbs, Richard; University of Oxford, Nuffield Department of Primary Care Health Sciences<br>Perera, Rafael; University of Oxford, Primary Health Care<br>Sherlock, Julian; University of Oxford, Nuffield Department of Primary Care Health Sciences; University of Surrey, Department of Clinical and Experimental Medicine<br>Yonova , Ivelina; Royal College of General Practitioners, Research and Surveillance Centre<br>Bankhead, Clare; University of Oxford, Nuffield Department of Primary Care Health Sciences
Keywords:	PRIMARY CARE, ONCOLOGY, Health informatics < BIOTECHNOLOGY & BIOINFORMATICS

SCHOLARONE™
Manuscripts

I, the Submitting Author has the right to grant and does grant on behalf of all authors of the Work (as defined in the below author licence), an exclusive licence and/or a non-exclusive licence for contributions from authors who are: i) UK Crown employees; ii) where BMJ has agreed a CC-BY licence shall apply, and/or iii) in accordance with the terms applicable for US Federal Government officers or employees acting as part of their official duties; on a worldwide, perpetual, irrevocable, royalty-free basis to BMJ Publishing Group Ltd ("BMJ") its licensees and where the relevant Journal is co-owned by BMJ to the co-owners of the Journal, to publish the Work in this journal and any other BMJ products and to exploit all rights, as set out in our licence.

The Submitting Author accepts and understands that any supply made under these terms is made by BMJ to the Submitting Author unless you are acting as an employee on behalf of your employer or a postgraduate student of an affiliated institution which is paying any applicable article publishing charge ("APC") for Open Access articles. Where the Submitting Author wishes to make the Work available on an Open Access basis (and intends to pay the relevant APC), the terms of reuse of such Open Access shall be governed by a Creative Commons licence – details of these licences and which Creative Commons licence will apply to this Work are set out in our licence referred to above.

Other than as permitted in any relevant BMJ Author's Self Archiving Policies, I confirm this Work has not been accepted for publication elsewhere, is not being considered for publication elsewhere and does not duplicate material already published. I confirm all authors consent to publication of this Work and authorise the granting of this licence.

For peer review only

CASNET2: Evaluation of an Electronic Safety Netting (E-SN) cancer toolkit for the primary care electronic health record: protocol for a pragmatic stepped-wedge RCT

Authors

Dr Susannah Fleming[1], Dr Brian D. Nicholson[1], Dr Afsana Bhuiya[2], Prof Simon de Lusignan[1,3,4], Dr Yasemin Hirst[5], Prof F.D. Richard Hobbs[1], Prof Rafael Perera-Salazar[1], Julian Sherlock[1,3], Ivelina Yonova[1,3,4], Prof Clare R. Bankhead[1]

Affiliations

[1]*Nuffield Department of Primary Care Health Sciences, University of Oxford*
Susannah Fleming, Senior Quantitative Researcher, susannah.fleming@phc.ox.ac.uk
Brian D Nicholson, Academic Clinical Lecturer, brian.nicholson@phc.ox.ac.uk
F.D. Richard Hobbs, Nuffield Professor of Primary Care, richard.hobbs@phc.ox.ac.uk
Rafael Perera-Salazar, Professor of Medical Statistics, rafael.perera@phc.ox.ac.uk
Clare R. Bankhead, Associate Professor, clare.bankhead@phc.ox.ac.uk

[1]*Nuffield Department of Primary Care Health Sciences, University of Oxford*
[3]*Department of Clinical and Experimental Medicine, University of Surrey, Guildford, GU2 7XH*
[4]*Royal College of General Practitioners Research and Surveillance Centre, Euston Square, London*
Simon de Lusignan, Professor of Primary Care and Clinical Informatics, simon.delusignan@phc.ox.ac.uk
Ivelina Yonova, Project Manager, ivelina.yonova@phc.ox.ac.uk

[1]*Nuffield Department of Primary Care Health Sciences, University of Oxford*
[3]*Department of Clinical and Experimental Medicine, University of Surrey, Guildford, GU2 7XH*
Julian Sherlock, SQL Developer, julian.sherlock@phc.ox.ac.uk

[2]*North Central and East London (NCEL) cancer alliance*
Afsana Bhuiya, Joint Chief Medical Officer, afsana.bhuiya1@nhs.net

[5]*Research Department of Behavioural Science and Health, University College London*
Yasemin Hirst, Research Associate, y.hirst@ucl.ac.uk

Corresponding Author

Brian D Nicholson, Academic Clinical Lecturer, brian.nicholson@phc.ox.ac.uk
Nuffield Department of Primary Care Health Sciences, University of Oxford

For peer review only

Word count: 3992 words

For peer review only

Abstract (303 words)

Introduction

Safety-netting in primary care is best practice in cancer diagnosis, ensuring that patients are followed-up until symptoms are explained or have resolved. Currently, clinicians use haphazard and often manual solutions. The ubiquitous use of electronic health records (EHR) provides an opportunity to standardise safety-netting practices.

A new Electronic Safety-netting (E-SN) toolkit, has been introduced to provide systematic ways to track and follow-up patients. We will evaluate the effectiveness of this toolkit, which is already embedded in a major primary care clinical system in England (EMIS-Web).

Methods and analysis

We will conduct a stepped-wedge cluster RCT in 60 general practices within the RCGP Research and Surveillance Centre (RSC) network. Groups of 10 practices will be randomised into the active phase at 2-monthly intervals over 12 months. All practices will be activated for at least 2 months. The primary outcome is the primary care interval measured as days between 1st recorded symptoms of cancer (within the year prior to diagnosis) and subsequent referral to secondary care. Other outcomes include rates of referrals via the UK's very urgent 2 week wait, urgent and routine pathways, and the rate of direct access cancer investigation.

Analysis of the clustered stepped-wedge design will model associations using a fixed effect for intervention condition of the cluster at each time step, a fixed effect for time and other covariates, and then include a random effect for practice and for patient to account for correlation between observations from the same centre and from the same participant.

Ethics and dissemination

Ethical approval has been obtained (19/NW/0692). Results will be disseminated in peer-reviewed journals and conferences, and sent to participating practices. They will be published on the University of Oxford Nuffield Department of Primary Care and RCGP RSC websites.

Registration details

ISRCTN registry trial ID ISRCTN15913081.

Keywords

Primary Care, Cancer, Diagnostic Safety, Clinical Informatics, Randomised Controlled Trial.

Article Summary

- The safety-netting toolkit being evaluated is already implemented and integrated in the EMIS Web, a brand of electronic health record (EHR), but is rarely utilised

For peer review only

- Data collection will be carried out primarily through existing automated links between the practice EHR and the RCGP RSC, surveillance system, thereby limiting the additional research workload for participating practices
- We are reliant on the cancer diagnosis, generally made in specialist care, being recorded in the primary care EHR system in a consistent and timely manner.

Introduction

Background

Safety-netting is regarded as "best practice" in cancer diagnosis in primary care.(1) It aims to ensure patients do not drop through the healthcare net but are followed-up until symptoms are explained.(2) Our research highlights an absence of evidence on how best to safety-net, especially with patients with non-specific cancer symptoms.(1) Expert consensus, international survey data and interviews with GPs and patients show that effective patient communication, shared decision making and improved clinical systems are needed to ensure that tests and referrals are followed-up and recurrent consultations are identified in patients with unexplained symptoms.(3-5) To achieve this, significant improvements in electronic health record (EHR) utilisation, particularly data quality, are required, by integrating information and communication technology with clinical care.(6-8)

Whilst there are clear guidance and recommendations for safety netting in primary care,(9) successful implementation of these recommendations rely on resources available at general practices. Universally accessible NHS fail-safes do not exist to ensure tests are conducted, returned and reconciled.(10) Confusion exists about which staff member is responsible for test communication.(11) Patients can be unaware of their responsibility to follow up investigations and referrals, assuming "no news is good news," and taking no action if they do not feel better or develop new symptoms.(12, 13) The success of a systems-based approach to safety-netting is jeopardised by inadequate administrative processes and marked variation in approaches to follow-up.(14) EHR based interventions show promise: trials in the United States (US) of electronic prompts increased the proportion of patients with cancer symptoms who receive follow-up.(8, 15-17) However, despite reporting enthusiasm for new initiatives, GPs do not always engage with new information technology, and this driven in part by social and technical factors, such as pop-up fatigue and information overload and being under-resourced (13, 14, 18-21).

An electronic safety-netting toolkit (E-SN toolkit) has been developed through consultation with GPs in the UK and was embedded within one of the major clinical systems in England - Egerton Medical Information System (EMIS) Web in May 2018. The toolkit is designed to replace existing verbal or paper methods of safety netting by providing effective means for tracking and follow-up by administrative staff. The E-SN toolkit proposes a rigorous, robust, traceable and auditable pro-active approach to tracking patients. It is designed to allow clinical data to be entered using templates, and diary entries to be generated (time reminders to check an action has been completed). Using diary entries, users can effectively follow up test requests, referrals, and non-specific but concerning symptoms. For instance, outstanding actions appear as "Alert Flags" to identify incomplete diary entries and can be collated. Although the E-SN toolkit is currently available to general practices using EMIS, practices have to proactively turn it on to implement it in their current practice.

This study aims to evaluate the effectiveness of an EHR safety-netting toolkit (E-SN toolkit) for use in primary care with patients with symptoms of cancer.

For peer review only - http://bmjopen.bmj.com/site/about/guidelines.xhtml

Study design

The study will employ a stepped wedge cluster-randomised controlled trial (SW-CRCT) randomising clusters, i.e. general practices, in blocks of 10 to the timing of activation of the E-SN toolkit. General practices will cross-over in these blocks to the activated phase every two months (Figure 1). The study will compare patient's primary care interval data for cancer diagnoses pre and post E-SN toolkit activation at all participating practices. The stepped-wedge design will ensure that time-related confounders such as seasonal variation should be accounted for.

This paper describes the protocol dated 7 October 2019 (version 1.5). Core trial information is given in Table 1.

The anticipated length of the study is 18 months. This consists of 3 months recruitment, followed by a 12 month period during which time the intervention will be introduced, and 3 months for analysis.

Methods and analyses

Outcomes and outcome definitions

Primary outcome: primary care interval for cancer diagnoses

The primary care interval is defined as the number of days between the first recorded symptoms of cancer (within the year prior to diagnosis) and subsequent referral for secondary cancer care. In line with published research and guidelines on diagnostic intervals, we will search the patient record for all patients with a cancer diagnosis for the year prior to diagnosis: one year is a trade-off between misattributing unrelated symptoms occurring more than a year before and missing symptoms of relevance by restricting to a shorter period.(22, 23)

Secondary Outcomes

Full details of the secondary outcomes are given in Table 2 and Figure 2.

Setting and participants

The study will be carried out in 60 general practices that contribute data to the RCGP Research and Surveillance Centre (RSC) Network (24, 25) and use the EMIS Web EHR system. The RCGP RSC includes general practices in England.

Inclusion and exclusion criteria

The inclusion criteria for general practices are as follows:

- Practice is actively contributing data to the RCGP RSC
- Utilises EMIS EHR system
- Data available for the previous 24 months

The exclusion criteria for general practices are:

- Practices that express an interest, but are not fully set up for data extraction
- Any practice already deploying the E-SN toolkit

For peer review only

Recruitment

The RCGP RSC network will identify potentially eligible practices and circulate the details of the study before and during the recruitment period. Practices will also be approached directly by the RCGP RSC Practice Liaison Officers (PLOs), who regularly visit practices to monitor their data quality and inform about current open studies. Expressions of Interest (EOI) will be obtained from all interested practices. An additional 12 practices will be identified to account for potential drop-outs during the study. All practices will be recruited at the start of the study prior to randomisation and implementation of the intervention.

General practices will receive reimbursement of up to £500 per practice for participation in the 12 month stepped-wedge intervention study.

Randomisation and blinding

The eligible practices will be ranked according to their list size from smallest to the largest by the RCGP RSC. These will then be stratified into 10 strata (by list size) such that each strata contains the same number of practices. This is based on the allocation of 10 practices per step. Practices will be randomised in blocks of 10 to the timing of activation of the E-SN toolkit and will cross-over in these blocks to the activated phase every two months. Therefore practices will contribute between 2 months and 12 months of E-SN toolkit-activated time (Figure 1).

A statistician (Rafael Perera, Nuffield Department of Primary Care Health Sciences, University of Oxford) who is independent to the intervention development and implementation will produce a stratified randomisation schedule so that within each strata, practices are randomly allocated to each of the 6 steps, with some replacement practices. The random sequence will be generated using R software. The allocation will be undertaken for all practices at the same time. Where replacement practices are required, these will be taken from the same strata where possible. If no replacement practices from the same strata are available a coin toss will determine whether the next highest or lowest strata will be used to provide replacement practices.

Given the practice change nature of the intervention, clinicians and practice managers will be aware when their practice has switched to the intervention period. Consulting patients providing outcome data will not be informed of the experimental nature of the E-SN Toolkit activation and therefore will be blind to the stage of study occurring in the practice they attended. Study personnel involved in extracting outcome data will be blind to the allocated order of the delivery of the intervention across the practices. All data management of extracted data to calculate the outcome measures will be conducted blinded to the timing of switching to intervention. Similar methods have been used in other implementation SW-CRTs.(26)

Intervention

The intervention consists of activation and the implementation of the E-SN toolkit at participating practices. All practices will receive training in the use of the E-SN toolkit from RCGP PSC Practice Liaison Officers prior to their switching date. Clinicians will be able to use the E-SN templates at their own discretion during the active period, with no requirements on which patients should or should not receive safety netting. Clinical care of patients will continue as normal throughout the study.

For peer review only

The E-SN toolkit uses a templates to track cancer events like referrals, direct access tests and monitoring of low risk through read codes attached to diary entries. Expired diary entries would be identified using automated searches and actioned by the administration lead as appropriate. If an event was complete e.g. normal scan results done in 2 weeks and result back - then the diary entry is closed, resolving the episode. The E-SN toolkit has extra features such as popup alerts to remind any user there is an open diary entry and also allows the E-SN toolkit to popup automatically if a relevant code is used in the clinical records.

Further details and demonstration video are available from: https://www.uclh.nhs.uk/OurServices/ServiceA-Z/Cancer/NCV/MICa/Pages/Primarycareimprovement.aspx

Practices will all be encouraged to adhere to the schedule for switching and will receive part-payment for study initiation and then full payment when they adhere to the activation schedule.

Data extraction

Data extractions from all participating practices will correspond to two major time points: at the start of the introduction to the stepped wedge implementation of the E-SN toolkit, and at the end of the stepped wedge period (12 months later). At these two time points, consultation data from the participating practices will be obtained for the previous 24 months. Data from the previous 24 months is required to identify cancers detected in 12 months prior to start, plus the first recorded symptom of cancer during the primary care interval, allowing for this interval to cover up to a year prior to diagnosis.

Records from all patients who are adults aged 18 years and above, registered at participating practices and who have not opted out of sharing data for research purposes will be extracted. Pseudonymised patient information will include: demographic information of age, sex, dates of clinical consultations; coded consultation data for symptoms, diagnoses, tests ordered, and referrals made within the consultation records. Where a definitive diagnosis has been made (for cancer and non-cancer conditions), the clinical features recorded in the year prior to diagnosis will be captured. These actions will be achieved by extraction of the electronic health record rather than by hand searching notes.

Practices will be asked to search for open diary entries every week during the intervention period, and to download and save these to an Excel spreadsheet (locally). The central study research team will use these spreadsheets to track which diary entries are closed during the course of the intervention.

Data management

The principal data source for the this study are pseudonymised routinely collected care data extracted from general practices of the RCGP RSC network's database; there are over 500 general practices in this network. All general practices in the UK use an EHR to maintain patient medical records. Data are entered into a patient's computerised medical system as coded data or free text. The RCGP RSC extract coded data, i.e. where the clinician codes a disease or symptom into their EHR system.(27)

The RCGP RSC has no role in updating clinical data recorded by clinicians as part of their consultation and care. However, the RCGP RSC practices do received focussed feedback about data quality around its surveillance function, influenza other infections, vaccine update and effectiveness. This is done via team of PLOs and a dashboard.(28, 29)

For peer review only - http://bmjopen.bmj.com/site/about/guidelines.xhtml

RCGP RSC maintains an auditable trail for all the stages of data processing to ensure the quality of data are not compromised by the processing. For example, checking the prevalence of certain conditions and outliers revealed by the data is consistent with those reported in the literature. The standard operating procedures for data extraction and data processing, and data access are available from https://clininf.eu/index.php/information-governance/.

Discontinuations and withdrawals from the study

Each practice has the right to withdraw from the study at any time. Data from withdrawn practices will be included in analyses up to the point of withdrawal, unless they indicate that they wish to withdraw previously collected data from analysis.

Sample size

Practice lists sizes within the RCGP RSC are approximately 10,000. In England diagnosis rate of new cancer was 523/100,000 per year (2014/15).(30) Therefore we could expect 53 new cancers per year per practice. Therefore, in each 2 month step there would be 8 - 9 cancers per cluster.

The median primary care interval between first presentation and specialist referral(22, 31) is 5 days, interquartile range of 0-27.(32) Some cancers present with clear red flag symptoms leading to immediate specialist referral. Presentations of vague symptoms such as weight loss are less likely to be immediately referred and may benefit from using the safety-netting template. This symptom is associated with several cancers such as prostate, colorectal, lung, gastro-oesophageal, and pancreatic.(33) The median primary care interval for lung cancer is 14 days (interquartile range 2-45).(32) Using the *steppedwedge, detectable difference incomplete(1)* command in Stata 14 showed that with the design in Figure 1 and 60 practices we would be able to detect a difference of 2 days with 80% power.

Currently approximately 19% of cancers are diagnosed following an emergency presentation.(34) With 9 cancers per step per cluster we would be powered to detect a difference of 5%.

Under another scenario of considering primary care intervals towards the 90th centile of 60 days, with 60 practices, entering the stepped wedge design in 6 steps we would be able to detect a minimal difference of 13 days. However, if we consider that these patients with longer delays are in the 90th centile, then instead of expected cancers per cluster per step of 9 – there would be around 1. This would allow us to detect a minimal difference of between 9 and 39 days dependent on the assumption of the distribution of the primary care delays.

Several scenarios are shown in Table 3, all based on 60 practices, entering in 6 blocks, with a 12 month pre-intervention period. In summary our main analysis will focus on all cancers, but we can conduct pre-specified subgroup analyses restricting to cancers that typically have longer delays.

Statistical analysis

In patients with a diagnosis of cancer we will calculate the primary outcome of primary care interval (time between first recorded symptom and referral), proportion diagnosed after emergency presentation and the recorded diagnosis. Amongst consulting patients we will calculate the rate of direct access cancer investigation and rates of referrals via 2 week wait, urgent and routine pathways, as well as the number of consultations during the primary care interval, and the template activation rate.

For peer review only

Regarding the analysis of the stepped-wedge design and the effect of correlation of observations within clusters, will model the association using a fixed effect for the intervention condition of the cluster at each time step, a fixed effect for time and other covariates (e.g. changes in cancer guidelines), and then include a random effect for practice and a random effect for patient to account for correlation of the observations from the same centre and from the same participant.

Analyses will include all patients registered at participating practices. Practices that withdraw their agreement to participate will be included in analyses up to the point of withdrawal, unless they indicate that they wish to withdraw previously collected data from analysis.

Our primary analysis will be carried out on an intention to treat principle. Therefore, if any practice does not switch on the E-SN toolkit at the correct time for their cluster, we will carry out the analysis under the assumption that the E-SN toolkit was switched on at the correct time.

The primary care interval is defined from the presence of symptoms and referral in the primary care record. Missing data for these variables in the record will be interpreted as the absence of symptoms or referral (respectively). Extracted data from the primary care record will be limited to the study period plus the preceding year, so symptoms recorded prior to this will not be visible to the research team.

The study will not have a formal data monitoring committee as patients will be receiving standard care through their GP, and all data will be extracted from routinely collected clinical notes.

Planned subgroups

Where applicable, subgroups will be:

- Patients in whom an E-SN toolkit entry was completed
- Patients diagnosed with cancer

Data display and reporting

We will combine or suppress any cells with small numbers (under 5) of observations to prevent any potential identification during the reporting of the results.

Patient and public involvement

We have recruited an experienced PPI representative to sit on the project steering committee. She has been involved in planning how PPI involvement will best fit in with the project, and will attend steering committee meetings to discuss project progress, as well as co-chairing workshops with patient representatives. We anticipate involving five additional patient representatives. The PPI collaborators will be involved in interpreting the findings of the study and identifying outcomes that are of greatest importance to patients. This will allow them to consider whether there would be a beneficial effect in terms of patient perspectives, even if the E-SN Toolkit caused only a small change in outcomes.

Ethics and dissemination

Informed consent

Practices that are randomised to take part in the study will be provided with a welcome pack by their RCGP RSC PLO team. The pack includes a practice information leaflet and practice poster, a copy of the protocol, and a copy of the ethical approval documentation, providing the practice with detailed information about the exact nature of the study; study requirements; the implications and constraints

For peer review only

of the protocol; and any risks involved in taking part. Site agreements will be in place with each practice and it will be clearly stated that the practice is free to withdraw from the study at any time for any reason, without affecting their legal rights, and with no obligation to give the reason for withdrawal. Signing a site agreement will form consent for a practice to take part in the study.

No direct or active involvement will be required from consulting patients and we will not be seeking individual patient consent. The rationale for obtaining agreement at the cluster (practice) level is that the activation of the Toolkit will be through the EMIS Web software system and health care practitioners are the intended recipient of the intervention.(35, 36)

Patients who have opted out of sharing data are not processed by the RCGP RSC and therefore will not be accessed the research team and their data will not be extracted. RCGP RSC does keep a count of opt-out patients per practice as this is needed to interpret rates collected for surveillance, this runs at around 2% of the registered population.

A patient notification in the form of a poster will be displayed in all participating practices giving patients information about the study and how to opt out of data sharing. Outcome data will be extracted from the EHR by the SQL developer and provided in a pseudonymised form to the analysis team.

Safety reporting

As patients remain under their GP's care throughout the study, and serious adverse events such as death and hospitalisation unrelated to the study are expected in this patient group, no formal monitoring of serious adverse events will be carried out.

Quality assurance

The study may be monitored, or audited in accordance with the current approved protocol, GCP, relevant regulations and standard operating procedures by responsible individuals from the sponsor and the NHS trusts in which it is being carried out.

Ethical and regulatory considerations

Ethical approval has been obtained from the North West – Greater Manchester West NHS Research Ethics Committee (REC Reference 19/NW/0692). We have also obtained Health Research Authority (HRA) approval to carry out the study in the NHS. The study sponsor is the University of Oxford, UK (ctrg@admin.ox.ac.uk). All substantial amendments to the protocol will be submitted to the sponsor, the ethics committee, and the HRA, and, where necessary, their approval will be obtained.

The Investigator will ensure that this study is conducted in accordance with relevant regulations and with Good Clinical Practice. The University has a specialist insurance policy in place which would operate in the event of any participant suffering harm as a result of their involvement in the research (Newline Underwriting Management Ltd, at Lloyd's of London). NHS indemnity operates in respect of the clinical treatment that is provided. All participants will continue to receive NHS care during and after the study.

Patient confidentiality

The study staff will ensure that the practices' patients' anonymity is maintained. The practice patients will be identified only by an ID number on all study documents and any electronic database.

All documents will be stored securely and only accessible by study staff and authorised personnel. The study will comply with the UK General Data Protection Regulation (GDPR) and Data Protection Act 2018,

For peer review only

which require data to be anonymised as soon as it is practical to do so. Pseudonymisation will be carried out using the RCGP RSC standard processes and will ensure that it is not possible for research staff to link study data with data from other sources. Although multiple items will be extracted from individual clinical records, we have taken care to minimise the number of data items/variables made available for this analysis, for example using age rather than date of birth. RCGP RSC apply a process of statistical disclosure control (SDC) to ensure that individuals cannot be identified, even from aggregate data. For example, data might have to be exported by 5- or 10-year age-bands if there were small numbers in an individual year of birth.

Data are held on a protected by a firewall secure server at the University of Surrey, currently acting as the RCGP RSC's data and analysis hub. All in-bounded connections are blocked, but out-bounded connections are allowed upon approval by a senior staff member.

The secure server is managed by the Clinical Informatics and Health Outcomes Research Group, and will be moved to the University of Oxford where the Group is based. It meets the requirements of NHS Digital's Data Security and Privacy (DSP) toolkit.

Study management

The study will be overseen by three groups (see Table 4 for composition and roles of members.) Day-to-day management of the study will be primarily carried out by a core group of three researchers, meeting weekly to discuss any ongoing issues, with other members of the management group brought in to advise as necessary.

The study management group will meet quarterly and will provide pragmatic scientific and methodological support, management oversight, and will participate in dissemination activities and planning of future funding applications.

The study advisory board will meet quarterly and will focus on strategic oversight and progression reviews, and dissemination activities.

Dissemination

The primary results of the study will be published in a peer-reviewed journal publication. Additional dissemination may take place via peer-reviewed conference presentations, and additional journal publications. Participating general practices will receive a report of the main findings, and results will also be disseminated on both the University of Oxford Nuffield Department of Primary Care and RCGP RSC websites. Patients will not be directly informed of the results, but will be able to access results on the internet if they so wish.

Authorship of all publications will be determined in accordance with the International Committee of Medical Journal Editors (ICMJE) guidelines.

The final trial dataset will consist of a large quantity of pseudonymised participant-level data extracted from clinical records. Due to the potentially sensitive nature of this data, and to ensure compliance with relevant data protection law, this data will only be made accessible to the subgroup within the research team who will be carrying out the analysis, and there are no plans for granting public access to the data.

The full protocol will be made available as part of the study ISRCTN registration details following publication of this paper. Statistical code will be available from the authors on request.

For peer review only

Author Statement

CRB, BDN, RH, RP, AB, YH, SdL, IY and JS contributed to writing the original grant application, on which the protocol was based. SF, CRB and BDN wrote the original protocol, with contributions from RP, AB, IY and SdL. SF, CRB, and BDN wrote the paper. All authors commented on the paper and approved the final version.

Funding

This work is supported by Cancer Research UK, Early Diagnosis Advisory Group (EDAG) grant number C48270/A27880. Neither the funder nor the sponsor will have any role in the study design, data collection, management, analysis or interpretation of data, writing of reports, or the decision to submit reports for publication. CRB is supported by the NIHR Oxford Biomedical Research Centre and the Applied Research Collaborative, Oxford. AB is funded by the NCEL Cancer Alliance. YH is funded by a Cancer Research UK Early Detection Grant (C38463/A26726). FDRH acknowledges his part-funding from the National Institute for Health Research (NIHR) School for Primary Care Research, the NIHR Collaboration for Leadership in Health Research and Care (CLARHC) Oxford, the NIHR Oxford Biomedical Research Centre (BRC), and the NIHR Oxford Medtech and In-Vitro Diagnostics Co-operative (MIC). SdeL has received commercial funding through his university for research funded by Seqirus, GSK, Lilly, Novo Nordisk, Astra Zenaca and Takeda principally for unrelated research in the areas of diabetes and vaccination. RP also received funding from the National Institute for Health Research (NIHR) Oxford Biomedical Research Centre, the NIHR Oxford Medtech and In-Vitro Diagnostics Co-operative, the NIHR Applied Research Collaboration Oxford and Thames Valley, and the Oxford Martin School.

Conflicts of interests

AB is the innovator of the E-SN toolkit and locally promotes the toolkit for the purposes of cancer care along its pathway. SF is a member of the National Body Temperature Measurement Group. SdeL is Director of the RCGP, as part of his academic work. CRB, YH, FDRH, BDN, RP, JS, and IY have no competing interests.

For peer review only

References

1. Nicholson BD, Mant D, Bankhead C. Can safety-netting improve cancer detection in patients with vague symptoms? BMJ. 2016;355:i5515.
2. Lyratzopoulos G, Vedsted P, Singh H. Understanding missed opportunities for more timely diagnosis of cancer in symptomatic patients after presentation. Br J Cancer. 2015;112 Suppl 1:S84-91.
3. Bankhead C, Heneghan C, Hewitson P, Thompson M. Safety netting to improve early cancer diagnosis in primary care: development of consensus guidelines. Department of Primary Health Care: University of Oxford; 2011.
4. Evans J, Ziebland S, MacArtney JI, Bankhead CR, Rose PW, Nicholson BD. GPs' understanding and practice of safety netting for potential cancer presentations: a qualitative study in primary care. Br J Gen Pract. 2018;68(672):e505-e11.
5. Nicholson BD, Goyder CR, Bankhead CR, Toftegaard BS, Rose PW, Thulesius H, et al. Responsibility for follow-up during the diagnostic process in primary care: a secondary analysis of International Cancer Benchmarking Partnership data. Br J Gen Pract. 2018;68(670):e323-e32.
6. Singh H, Spitzmueller C, Petersen NJ, Sawhney MK, Smith MW, Murphy DR, et al. Primary care practitioners' views on test result management in EHR-enabled health systems: a national survey. J Am Med Inform Assoc. 2013;20(4):727-35.
7. Callen JL, Westbrook JI, Georgiou A, Li J. Failure to follow-up test results for ambulatory patients: a systematic review. J Gen Intern Med. 2012;27(10):1334-48.
8. Improving diagnosis in health care [press release]. Washington, DC: The National Academies Press,2015.
9. NHS England Patient Safety Domain. Standards for the communication of patient diagnostic test results on

discharge from hospital. London: NHS England; 2016.
10. Litchfield I, Bentham L, Lilford R, McManus RJ, Hill A, Greenfield S. Test result communication in primary care: a survey of current practice. BMJ Qual Saf. 2015;24(11):691-9.
11. Litchfield IJ, Bentham LM, Lilford RJ, Greenfield SM. Test result communication in primary care: clinical and office staff perspectives. Fam Pract. 2014;31(5):592-7.
12. Singh H, Sittig DF. Were my diagnosis and treatment correct? No news is not necessarily good news. J Gen Intern Med. 2014;29(8):1087-9.
13. Litchfield IJ, Bentham LM, Lilford RJ, McManus RJ, Greenfield SM. Patient perspectives on test result communication in primary care: a qualitative study. Br J Gen Pract. 2015;65(632):e133-40.
14. Litchfield I, Bentham L, Hill A, McManus RJ, Lilford R, Greenfield S. Routine failures in the process for blood testing and the communication of results to patients in primary care in the UK: a qualitative exploration of patient and provider perspectives. BMJ Qual Saf. 2015;24(11):681-90.
15. Murphy DR, Laxmisan A, Reis BA, Thomas EJ, Esquivel A, Forjuoh SN, et al. Electronic health record-based triggers to detect potential delays in cancer diagnosis. BMJ Qual Saf. 2014;23(1):8-16.
16. Murphy DR, Thomas EJ, Meyer AN, Singh H. Development and Validation of Electronic Health Record-based Triggers to Detect Delays in Follow-up of Abnormal Lung Imaging Findings. Radiology. 2015;277(1):81-7.
17. Murphy DR, Wu L, Thomas EJ, Forjuoh SN, Meyer AN, Singh H. Electronic Trigger-Based Intervention to Reduce Delays in Diagnostic Evaluation for Cancer: A Cluster Randomized Controlled Trial. J Clin Oncol. 2015;33(31):3560-7.
18. Gross Z, Ozdilli J, Anastasiadis T, Ryan H. Using Cancer Decision Support Tools to support the early diagnosis of cancer 2017 [Available from: https://www.cancerresearchuk.org/sites/default/files/ace_cancer_decision_support_tools_final_report_v1.1_080517.pdf.
19. Legare F, Ratte S, Gravel K, Graham ID. Barriers and facilitators to implementing shared decision-making in clinical practice: update of a systematic review of health professionals' perceptions. Patient Educ Couns. 2008;73(3):526-35.

For peer review only

20. Usher-Smith J, Emery J, Hamilton W, Griffin SJ, Walter FM. Risk prediction tools for cancer in primary care. Br J Cancer. 2015;113(12):1645-50.
21. Murphy DR, Reis B, Kadiyala H, Hirani K, Sittig DF, Khan MM, et al. Electronic health record-based messages to primary care providers: valuable information or just noise? Arch Intern Med. 2012;172(3):283-5.
22. Weller D, Vedsted P, Rubin G, Walter FM, Emery J, Scott S, et al. The Aarhus statement: improving design and reporting of studies on early cancer diagnosis. Br J Cancer. 2012;106(7):1262-7.
23. Neal RD, Din NU, Hamilton W, Ukoumunne OC, Carter B, Stapley S, et al. Comparison of cancer diagnostic intervals before and after implementation of NICE guidelines: analysis of data from the UK General Practice Research Database. Br J Cancer. 2014;110(3):584-92.
24. Correa A, Hinton W, McGovern A, van Vlymen J, Yonova I, Jones S, et al. Royal College of General Practitioners Research and Surveillance Centre (RCGP RSC) sentinel network: a cohort profile. BMJ Open. 2016;6(4):e011092.
25. de Lusignan S, Correa A, Smith GE, Yonova I, Pebody R, Ferreira F, et al. RCGP Research and Surveillance Centre: 50 years' surveillance of influenza, infections, and respiratory conditions. Br J Gen Pract. 2017;67(663):440-1.
26. Kingsland M, Doherty E, Anderson AE, Crooks K, Tully B, Tremain D, et al. A practice change intervention to improve antenatal care addressing alcohol consumption by women during pregnancy: research protocol for a randomised stepped-wedge cluster trial. Implement Sci. 2018;13(1):112.
27. de Lusignan S. Codes, classifications, terminologies and nomenclatures: definition, development and application in practice. Inform Prim Care. 2005;13(1):65-70.
28. Liyanage H, Williams J, Byford R, Pathirannehelage S, de Lusignan S. Near-Real Time Monitoring of Vaccine Uptake of Pregnant Women in a Primary Care Sentinel Network: Ontological Case Definition Across Heterogeneous Data Sources. Stud Health Technol Inform. 2019;264:1855-6.
29. Pathirannehelage S, Kumarapeli P, Byford R, Yonova I, Ferreira F, de Lusignan S. Uptake of a Dashboard Designed to Give Realtime Feedback to a Sentinel Network About Key Data Required for Influenza Vaccine Effectiveness Studies. Stud Health Technol Inform. 2018;247:161-5.
30. Public Health England. Cancer Services: Demographics, Screening and diagnostics 2018 [Available from: https://fingertips.phe.org.uk/profile/cancerservices/data#page/0/.
31. Koo MM, Hamilton W, Walter FM, Rubin GP, Lyratzopoulos G. Symptom Signatures and Diagnostic Timeliness in Cancer Patients: A Review of Current Evidence. Neoplasia. 2018;20(2):165-74.
32. Swann R, McPhail S, Witt J, Shand B, Abel GA, Hiom S, et al. Diagnosing cancer in primary care: results from the National Cancer Diagnosis Audit. Br J Gen Pract. 2018;68(666):e63-e72.
33. Nicholson BD, Hamilton W, O'Sullivan J, Aveyard P, Hobbs FR. Weight loss as a predictor of cancer in primary care: a systematic review and meta-analysis. Br J Gen Pract. 2018;68(670):e311-e22.
34. Public Health England. National Cancer Registration and Analysis Service: Cancer Outcome Metrics 2018 2018 [Available from: http://www.ncin.org.uk/cancer_type_and_topic_specific_work/topic_specific_work/cancer_outcome_metrics.
35. Dregan A, McDermott L, Ashworth M, McCann G, Charlton J, Yardley L, et al. Electronically-delivered, multi-component interventions to reduce unnecessary antibiotic prescribing in primary care. A cluster randomised trial using electronic health records (eCRT2). Health Technology Assessment. 2019;13/88/10.
36. Hutton JL. Are distinctive ethical principles required for cluster randomized controlled trials? Stat Med. 2001;20(3):473-88.

For peer review only

Figures and Tables

Figure 1. Stepped wedge design with 12 months pre-randomisation period

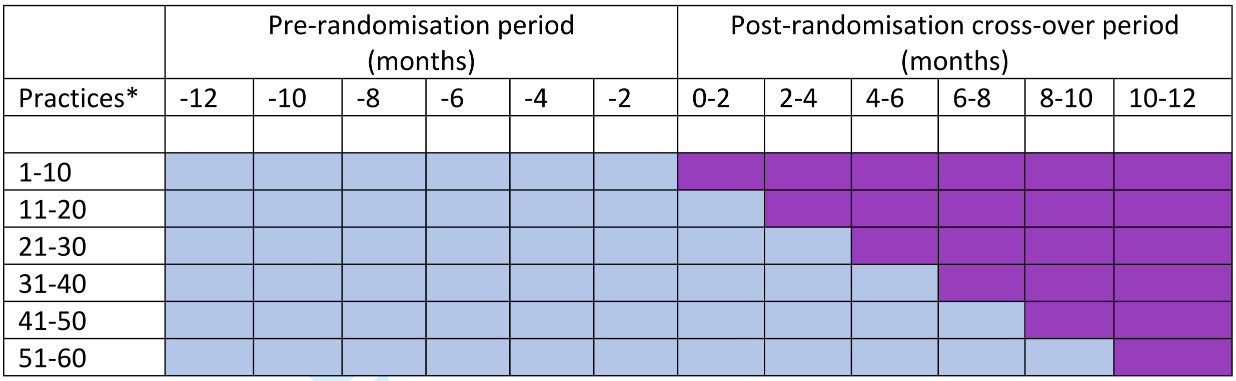

**Practices will be randomly allocated to the group and date of cross-over*

Pale blue cells represent inactive E-SN toolkit period
Purple cells represent active E-SN toolkit period

Figure 2. Diagram showing the definitions of time-based outcomes

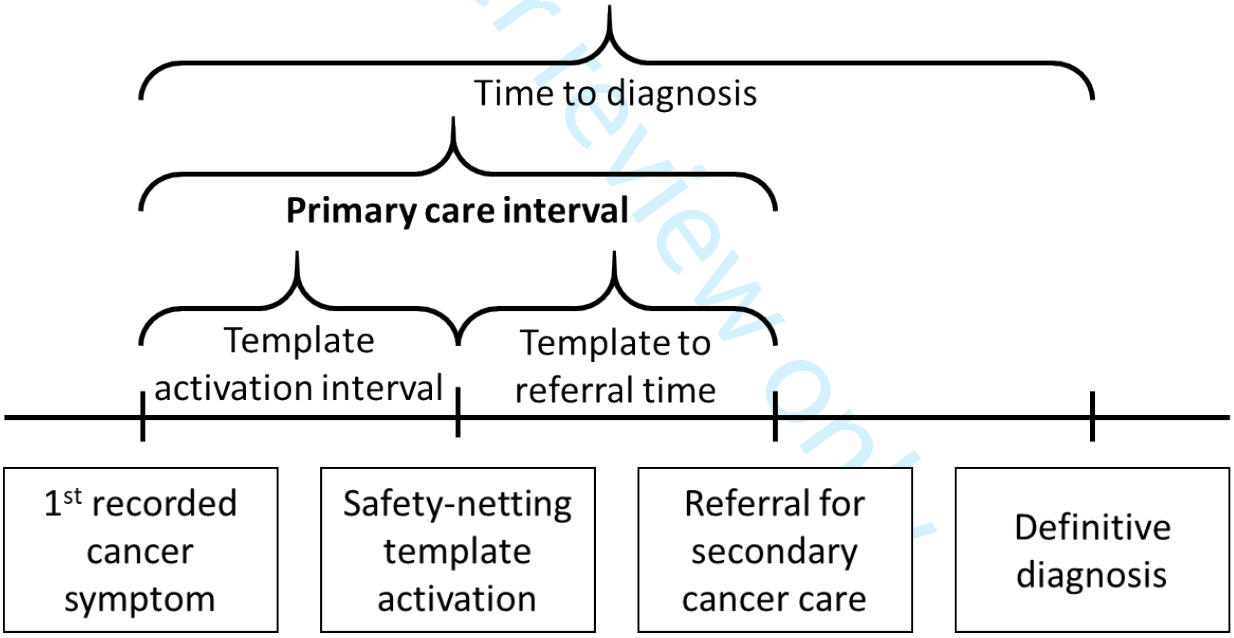

For peer review only

Table 1: WHO trial registration data set

Data category	Information
**Primary Registry and Trial Identifying Number**	ISRCTN: ISRCTN15913081
**Date of Registration in Primary Registry**	08/11/2019
**Secondary Identifying Numbers**	N/A
**Source(s) of Monetary or Material Support**	Cancer Research UK; Grant Codes: C48270/A27880
**Primary Sponsor**	University of Oxford
**Secondary Sponsor(s)**	None
**Contact for Public Queries**	susannah.fleming@phc.ox.ac.uk
**Contact for Scientific Queries**	clare.bankhead@phc.ox.ac.uk
**Public Title**	Testing an electronic safety netting system to help GPs follow up patients with worrying symptoms
**Scientific Title**	CASNET2: Evaluation of an e-safety netting cancer template in primary care: a pragmatic stepped-wedge RCT
**Countries of Recruitment**	United Kingdom
**Health Condition(s) or Problem(s) Studied**	E-safety netting (E-SN) toolkit
**Intervention(s)**	The researchers will recruit 60 general practices who are not currently using the E-SN toolkit, and randomise them in clusters (groups) of 10. Each cluster will have the E-SN toolkit turned on at a different time during the 12 months of the study. Once the E-SN toolkit is turned on, the GPs in the practice will be able to use it when caring for any patient they think would benefit from it, although it is expected that it will be of most use when treating patients with symptoms that might indicate cancer. The researchers will collect data from the electronic patient record system from the 12 months of the study and the 24 months before the start of the study to understand whether the introduction of the E-SN toolkit makes any difference to the diagnosis of cancer, and in particular to how quickly patients are diagnosed. The researchers will only extract records from patients who are over 18, and who have not opted out of the research.
**Key Inclusion and Exclusion Criteria**	GP practices will be eligible for inclusion under the following conditions:<br>1. They are actively contributing to the RCGP Research and Surveillance Centre database<br>2. They use the EMIS electronic health record system<br>3. They have data available for the previous 24 months<br>Within the participating practices, the researchers will seek to extract data from adult patients (aged over 18 years)<br><br>Exclusion criteria:<br>1. GP practices who are already using the E-SN toolkit will not be eligible for the study<br>2. The researchers will not extract data from any patient under 18, or from any patient who has opted out of data sharing for research purposes
**Study Type**	Other
**Date of First Enrollment**	25/11/2019
**Target Sample Size**	60
**Recruitment Status**	Not yet recruiting
**Primary Outcome(s)**	Primary care interval for cancer diagnoses measured as the time between the first recorded symptom of cancer and referral to secondary cancer care, during inactive and active E-SN phases
**Key Secondary Outcomes**	1. Proportion of cancers diagnosed after emergency presentation measured during inactive and active E-SN phases<br>2. Recorded new diagnoses in those who have a template activated, measured by cancer site and stage, and by non-cancer diagnosis, during the active E-SN stage<br>3. Total time to diagnosis measured from 1st recorded symptom to definitive diagnosis for all cancer diagnoses during the inactive and active E-SN phases and all diagnoses with template activation during the active E-SN phase<br>4. Number of GP consultations/patient between first record of symptom and cancer referral, measured during the inactive and active E-SN phase<br>5. Rates of patients completing direct access cancer investigations measured during the inactive and active E-SN phase<br>6. Rates of patients referred measured as 2-week wait, urgent, and routine, during the inactive and active E-SN phase<br>7. Timing of template activation within the primary care interval (from first symptom to referral) measured during the active E-SN phase<br>8. Template activation rate amongst consulting patients, both total and stratified by individual GP, measured during the active E-SN phase<br>9. The proportion of diary entries completed measured during the active E-SN phase

For peer review only - http://bmjopen.bmj.com/site/about/guidelines.xhtml

	10. The reason for template activation measured based on 20 high-level READ codes during the active E-SN phase<br>11. Symptoms leading to direct access to investigations measured during the active E-SN phase<br>12. Recorded vague symptoms in the template measured during the active E-SN phase<br>13. Demographic details of patients with activated templates measured during the active E-SN phase<br>14. GP type completing template (e.g. partner, locum, trainee) measured during the active E-SN phase<br>15. Diagnostic codes in patients with activated templates measured during the active E-SN phase

Table 2. Outcomes, measures, and time periods of measurement for primary and secondary outcomes

Outcome	Measure	Inactive Study Period	Active Study Period	Template activations only
**Primary outcome**				
**Primary care interval for cancer diagnoses**	Measured as the number of days between 1st recorded symptoms of cancer (within the year prior to diagnosis) and subsequent referral for secondary cancer care	X	X	
**Secondary outcomes**				
**Proportion of cancers diagnosed after emergency presentation**	Proportion of cancers for which diagnosis is made prior to referral, including following A&E or inpatient episodes. Where there is uncertainty regarding the route of diagnosis, the RCGP RSC network will contact the practice in an attempt to augment the data. Algorithms will also be developed to identify emergency presentations of cancer.	X	X	
**Recorded new diagnoses in those who have a template activated**	By cancer site and stage, and non-cancer diagnoses. Coded entries for all alternative diagnoses where the E-SN toolkit has been activated will be identified.		X	X
**Total time to diagnosis (from 1st recorded symptom to definitive diagnosis)**	Measured from 1st recorded symptom of cancer (within the previous year) to definitive diagnosis for all cancers diagnosed, and for all patients with an activated template	X	X	X (for non-cancer diagnoses)
**Number of GP consultations/patient between first record of symptom and cancer referral**	Measured as number of primary care consultations between the 1st recorded symptoms (within the year prior to diagnosis) and subsequent referral, per patient.	X	X	
**Rates of patients completing direct access cancer investigations**	Measured as the number of patients undergoing direct access cancer investigations (according to those specified in referral guidelines NG12) in each period divided by the person years of observation for that period.	X	X	
**Rates of patients referred for cancer**	Referrals rates via 2 week wait, urgent, and routine routes for all patients referred for specialist opinion to a secondary care cancer specialist	X	X	
**Timing of template activation**	Measured as the number of days between 1st recorded symptoms (within the year prior to diagnosis), and template activation and the number of days between template activation and subsequent referral.		X	X
**Template activation rate amongst consulting patients**	Measured as the number of patients with an activated template divided by the number of patients consulting, in each time period.<br>Both total rate and rate stratified by individual GP will be measured.		X	X
**Proportion of diary entries that were completed**	Measured as the number of diary entries that were completed divided by the number of diary entries that were opened.		X	X
**Reason for template activation**	The coded reasons for activating the template,<br>Based on 20 high level READ codes		X	X
**Symptoms leading to direct access to investigation**	All symptoms recorded in patients undergoing direct access cancer investigations	X	X	
**Recorded vague symptoms in the template**	All symptoms recorded within the template.		X	X
**Demographic details of patients with activated templates**	Age and sex of patients that had a template activated during the course of the trial.		X	X
**GP type completing templates**	Descriptive data on the type of GP that first activated the template (e.g. partner, locum, trainee)		X	X

**Diagnostic codes in patients with activated templates**	Diagnoses recorded after the activation of template.		X	X

Table 3. Scenarios for various assumptions used in the sample size calculations

Assumptions	All cancers (based on lung cancer)	All cancers	All cancers	All cancers	Restricting to only 90th centile delays	Restricting to only 90th centile delays
**Median (days)**	14	14	60	60	60	60
**Range (days)**	0-60	0 – 100	0 - 365	0 - 100	14 - 100	14 - 365
**N of cancers per step per cluster**	9	9	9	9	1	1
**Minimum detectable difference (days)**	2	5	13	4	9	39
**Notes**		Allowing greater range		Lower upper value for Primary Care Interval	Minimum set to median of all cancers	Min set to median of all cancers, but increased upper range

Table 4. Membership of groups overseeing the study

Member	Role	Day to day management	Management group	Advisory board
**Susannah Fleming**	Study co-ordinator	Yes	Yes	Yes
**Clare Bankhead**	PI	Yes	Yes	Yes
**Ivelina Yonova**	Project Manager	As necessary	Yes	Yes
**Brian Nicholson**	Co-PI	As necessary	Yes	Yes
**Simon de Lusignan**	Co-investigator		Yes	Yes
**Yasemin Hirst**	Co-investigator	As necessary	Yes	
**Afsana Bhuiya**	GP – Creator of E-SN toolkit	As necessary	Yes	
**Rafael Perera**	Statistician	As necessary	Yes	
**Julian Sherlock**	Programmer	As necessary	Yes	
**Sue Duncombe**	PPI representative	As necessary	Yes	Yes
**Jodie Moffatt**	Funder representative			Yes
**Rebecca Canning-Johns**	Independent statistician			Yes
**Richard Hobbs**	Co-investigator		Yes	
**Kathy Pritchard-Jones**	Advisor		Yes	

Appendices

Appendix A: List of study sites

Appendix B: Practice information sheet (RISP)

Appendix C: Patient poster

For peer review only

Appendix A: List of study sites

The list below is of NHS Local Clinical Research Networks within whose areas the general practices in the study will be located. At the time of publication, identification and recruitment of individual general practices for the study had not been completed. A complete list of individual sites will be available on request from the study team upon completion of study recruitment.

- North East and North Cumbria
- North West Coast
- Yorkshire and Humber
- Greater Manchester
- West Midlands
- West of England
- Thames Valley and South Midlands
- Eastern
- Kent, Surrey and Sussex
- Wessex
- South West Peninsula
- South London
- North West London

For peer review only

RISP
Research Information Sheet for Practices

**Study Title**	***CASNET2: Evaluation of an e-safety netting cancer template in primary care: a pragmatic stepped-wedge RCT***
**Principal Investigator(s) & Institution**	***Clare Bankhead, University of Oxford***
**NIHR Portfolio Ref**	**REC: TBC**<br>**IRAS ID: 269169**<br>**CPMS ID: TBC**
**Planned start date/ Duration of study recruitment at site**	Planned start date: 1 Sept 2019<br>12 months study duration – active period may be between 2 and 12 months depending on randomisation
**Study Funding Source**	Cancer Research UK
**Study Aim and Objectives**	The main aim of the study is to evaluate the E-safety netting (E-SN) toolkit, which is available as part of the EMIS electronic health record system. Our primary objective is to compare diagnostic outcomes for cancer (such as the time to diagnosis) during periods of time when the E-SN toolkit is turned off, compared with those during periods when the E-SN toolkit is turned on.<br>We also wish to compare consultation outcomes (such as the number of GP consultations before referral) during periods of time when the E-SN toolkit is turned off, compared with periods when the E-SN toolkit is turned on, to measure variation in the use of the E-SN toolkit between GP practices, and to describe the clinical situations for which the E-SN toolkit is used by GPs.<br>The E-SN toolkit is designed to assist with safety netting by providing a pro-active approach to tracking patients, ensuring that test requests, referrals and concerning symptoms are followed up in a timely manner.<br>We plan to recruit 60 general practices who are not currently using the E-SN toolkit, and randomise them in clusters (groups) of 10. Each cluster will have the E-SN toolkit turned on at a different time during the 12 months of the study. Once the E-SN toolkit is turned on, the GPs in the practice will be able to use it when treating any patient they think would benefit from it, although we expect that it will be of most useful when treating patients with symptoms that might indicate cancer.<br>We will collect data from the electronic patient record system from the 12 months of the study and the 24 months before the start of the study to understand whether the introduction of the E-SN toolkit makes any difference to the diagnosis of cancer, and particular to how quickly patients are diagnosed. We will only extract records from patients who are over 18, and who have not opted out of the research.

For peer review only - http://bmjopen.bmj.com/site/about/guidelines.xhtml

UNIVERSITY OF OXFORD · NUFFIELD DEPARTMENT OF PRIMARY CARE HEALTH SCIENCES · UNIVERSITY OF SURREY · RCGP Royal College of General Practitioners Research & Surveillance Centre

**Site target**	Not applicable as intervention is at practice rather than patient level
**Staff involvement at Practice**	General practitioners<br>Administrator
**Total funding paid**	Up to £500<br>• £100 for activating the template and attending the toolkit training provided by the study team<br>• £250 – for using the template as required during the study period<br>• £150 – to cover administrative time for keeping record of weekly searches and sending monthly reports to the study team
**Eligibility Criteria**	**Inclusion criteria:**<br>• Practice is actively contributing data to RCGP RSP<br>• Utilises EMIS electronic health records system<br>• Data available for the previous 24 months<br>• (Patients) Male or female, aged 18 years or above<br>**Exclusion criteria:**<br>• Practices that express an interest, but are not fully set up to start downloading data.<br>• Any practice already deploying the E-Safety netting toolkit within EMIS
**STUDY ACTIVITIES:**	
**Site Activities**	• Attend training in use of E-safety netting toolkit<br>• Activate E-safety netting toolkit in EMIS on date instructed by study team<br>• GPs to use E-safety netting toolkit in routine care when judged appropriate<br>• Administrator to perform weekly search in EMIS for open diary entries and download these to an Excel spreadsheet<br>• Downloaded Excel spreadsheets of open diary entries to be provided on a monthly basis to study team.
**Patient Activities**	None
**Recruitment data process**	Recruitment will be reported by the study team, so no recruitment upload is required by practices.
**Study specific training**	• GPs at participating sites will receive training in use of the E-safety netting toolkit.<br>• The study team will also provide training and support for administrators to assist with weekly searching for open diary entries.
**Possible risks**	• Use of the E-safety netting toolkit may cause a slight increase in overall consultation length, although this is expected to be of the order of tens of seconds<br>• Reduced trust in the health system due to patients becoming aware that their data is being used for research without explicit consent
**GDPR and confidentiality information**	Responsible members of the University of Oxford may be given access to data for monitoring and/or audit of the study to ensure that the research is complying with applicable regulations.<br>Data protection regulation requires that we state the legal basis for processing information about your practice and the patients registered there. In the case of research, this is 'a task in the public interest.' The University of Oxford is the data controller and is responsible for looking after this information and using it properly.<br>We will be using information from medical records in order to undertake this study. Patients who have opted not to share their information for disease surveillance will be respected by RCGP RSC and by the research team. The research team will not extract records of patients who have registered opt-out codes in the GP information system.

For peer review only - http://bmjopen.bmj.com/site/about/guidelines.xhtml

	Outcome data will be extracted from the EHR by the SQL developer and provided in a pseudonymised form to the analysis team.<br><br>You can find out more about how we use information from your practice and the patients registered there by contacting clare.bankhead@phc.ox.ac.uk or susannah.fleming@phc.ox.ac.uk
**Withdrawal from study**	Participation in the study is voluntary. If a practice chooses to withdraw from the study, data collected up to that point will be included in analyses. A practice may request upon withdrawal that data collected up to the point of withdrawal should not be used for analysis.<br>Practices who withdraw will receive the payment for activation and training, but payments for using the template and sending monthly reports of searches will be provided pro-rata for the period of time spent with the E-SN toolkit turned on before withdrawal.
**Making a complaint**	The University of Oxford, as Sponsor, has appropriate insurance in place in the unlikely event that you suffer any harm as a direct consequence of your participation in this study. NHS indemnity operates in respect of the clinical treatment which is provided.<br>If you wish to complain about any aspect of the way in which you have been approached or treated, or how your information is handled during the course of this study, you should contact Clare Bankhead on 01865 289351, or by email: clare.bankhead@phc.ox.ac.uk. Alternatively, you may contact the University of Oxford Clinical Trials and Research Governance (CTRG) office on 01865 616480, or the head of CTRG, email ctrg@admin.ox.ac.uk.
**Research Sponsor**	University of Oxford<br>ctrg@admin.ox.ac.uk
**General study contact**	Manasa Tripathy, Practice Liason Officer<br>manasa.tripathy@phc.ox.ac.uk

For peer review only - http://bmjopen.bmj.com/site/about/guidelines.xhtml

CASNET2 Study

RCGP

Research & Surveillance Centre Practice

This practice is taking part in the CASNET2 study. This is a study run by researchers at the University of Oxford looking at a new electronic safety netting tool which is part of the practice computer system. The tool helps GPs to make sure that patients get followed up if they have symptoms that might lead to a new diagnosis.

The researchers are hoping to find out whether the tool helps to reduce the time it takes for people to get a diagnosis. They will be using anonymised patient data from the practice computer system to find out how well the tool works. Using data in this way will not affect your privacy or care, and no-one will be able to identify you personally, but please speak to your GP if you have any concerns, or if you wish to opt out of the study.

For further information please visit http://www.rcgp.org.uk/rsc or contact:

Prof Simon de Lusignan
RSC Director
MedicalDirectorRSC@rcgp.org.uk

Dr Lucy Moore
Research Facilitator
practiceenquiries@phc.ox.ac.uk

Royal College of General Practitioners (RCGP) Research and Surveillance Centre (RSC)

For peer review only - http://bmjopen.bmj.com/site/about/guidelines.xhtml

SPIRIT

STANDARD PROTOCOL ITEMS: RECOMMENDATIONS FOR INTERVENTIONAL TRIALS

SPIRIT 2013 Checklist: Recommended items to address in a clinical trial protocol and related documents*

Section/item	Item No	Description
**Administrative information**		
Title	1	Descriptive title identifying the study design, population, interventions, and, if applicable, trial acronym
Trial registration	2a	Trial identifier and registry name. If not yet registered, name of intended registry
	2b	All items from the World Health Organization Trial Registration Data Set
Protocol version	3	Date and version identifier
Funding	4	Sources and types of financial, material, and other support
Roles and responsibilities	5a	Names, affiliations, and roles of protocol contributors
	5b	Name and contact information for the trial sponsor
	5c	Role of study sponsor and funders, if any, in study design; collection, management, analysis, and interpretation of data; writing of the report; and the decision to submit the report for publication, including whether they will have ultimate authority over any of these activities
	5d	Composition, roles, and responsibilities of the coordinating centre, steering committee, endpoint adjudication committee, data management team, and other individuals or groups overseeing the trial, if applicable (see Item 21a for data monitoring committee)
**Introduction**		
Background and rationale	6a	Description of research question and justification for undertaking the trial, including summary of relevant studies (published and unpublished) examining benefits and harms for each intervention
	6b	Explanation for choice of comparators
Objectives	7	Specific objectives or hypotheses
Trial design	8	Description of trial design including type of trial (eg, parallel group, crossover, factorial, single group), allocation ratio, and framework (eg, superiority, equivalence, noninferiority, exploratory)

For peer review only

Methods: Participants, interventions, and outcomes

Study setting	9	Description of study settings (eg, community clinic, academic hospital) and list of countries where data will be collected. Reference to where list of study sites can be obtained
Eligibility criteria	10	Inclusion and exclusion criteria for participants. If applicable, eligibility criteria for study centres and individuals who will perform the interventions (eg, surgeons, psychotherapists)
Interventions	11a	Interventions for each group with sufficient detail to allow replication, including how and when they will be administered
	11b	Criteria for discontinuing or modifying allocated interventions for a given trial participant (eg, drug dose change in response to harms, participant request, or improving/worsening disease)
	11c	Strategies to improve adherence to intervention protocols, and any procedures for monitoring adherence (eg, drug tablet return, laboratory tests)
	11d	Relevant concomitant care and interventions that are permitted or prohibited during the trial
Outcomes	12	Primary, secondary, and other outcomes, including the specific measurement variable (eg, systolic blood pressure), analysis metric (eg, change from baseline, final value, time to event), method of aggregation (eg, median, proportion), and time point for each outcome. Explanation of the clinical relevance of chosen efficacy and harm outcomes is strongly recommended
Participant timeline	13	Time schedule of enrolment, interventions (including any run-ins and washouts), assessments, and visits for participants. A schematic diagram is highly recommended (see Figure)
Sample size	14	Estimated number of participants needed to achieve study objectives and how it was determined, including clinical and statistical assumptions supporting any sample size calculations
Recruitment	15	Strategies for achieving adequate participant enrolment to reach target sample size

Methods: Assignment of interventions (for controlled trials)

Allocation:

Sequence generation	16a	Method of generating the allocation sequence (eg, computer-generated random numbers), and list of any factors for stratification. To reduce predictability of a random sequence, details of any planned restriction (eg, blocking) should be provided in a separate document that is unavailable to those who enrol participants or assign interventions

Allocation concealment mechanism	16b	Mechanism of implementing the allocation sequence (eg, central telephone; sequentially numbered, opaque, sealed envelopes), describing any steps to conceal the sequence until interventions are assigned
Implementation	16c	Who will generate the allocation sequence, who will enrol participants, and who will assign participants to interventions
Blinding (masking)	17a	Who will be blinded after assignment to interventions (eg, trial participants, care providers, outcome assessors, data analysts), and how
	17b	If blinded, circumstances under which unblinding is permissible, and procedure for revealing a participant's allocated intervention during the trial
**Methods: Data collection, management, and analysis**		
Data collection methods	18a	Plans for assessment and collection of outcome, baseline, and other trial data, including any related processes to promote data quality (eg, duplicate measurements, training of assessors) and a description of study instruments (eg, questionnaires, laboratory tests) along with their reliability and validity, if known. Reference to where data collection forms can be found, if not in the protocol
	18b	Plans to promote participant retention and complete follow-up, including list of any outcome data to be collected for participants who discontinue or deviate from intervention protocols
Data management	19	Plans for data entry, coding, security, and storage, including any related processes to promote data quality (eg, double data entry; range checks for data values). Reference to where details of data management procedures can be found, if not in the protocol
Statistical methods	20a	Statistical methods for analysing primary and secondary outcomes. Reference to where other details of the statistical analysis plan can be found, if not in the protocol
	20b	Methods for any additional analyses (eg, subgroup and adjusted analyses)
	20c	Definition of analysis population relating to protocol non-adherence (eg, as randomised analysis), and any statistical methods to handle missing data (eg, multiple imputation)
**Methods: Monitoring**		
Data monitoring	21a	Composition of data monitoring committee (DMC); summary of its role and reporting structure; statement of whether it is independent from the sponsor and competing interests; and reference to where further details about its charter can be found, if not in the protocol. Alternatively, an explanation of why a DMC is not needed

	21b	Description of any interim analyses and stopping guidelines, including who will have access to these interim results and make the final decision to terminate the trial
Harms	22	Plans for collecting, assessing, reporting, and managing solicited and spontaneously reported adverse events and other unintended effects of trial interventions or trial conduct
Auditing	23	Frequency and procedures for auditing trial conduct, if any, and whether the process will be independent from investigators and the sponsor
**Ethics and dissemination**		
Research ethics approval	24	Plans for seeking research ethics committee/institutional review board (REC/IRB) approval
Protocol amendments	25	Plans for communicating important protocol modifications (eg, changes to eligibility criteria, outcomes, analyses) to relevant parties (eg, investigators, REC/IRBs, trial participants, trial registries, journals, regulators)
Consent or assent	26a	Who will obtain informed consent or assent from potential trial participants or authorised surrogates, and how (see Item 32)
	26b	Additional consent provisions for collection and use of participant data and biological specimens in ancillary studies, if applicable
Confidentiality	27	How personal information about potential and enrolled participants will be collected, shared, and maintained in order to protect confidentiality before, during, and after the trial
Declaration of interests	28	Financial and other competing interests for principal investigators for the overall trial and each study site
Access to data	29	Statement of who will have access to the final trial dataset, and disclosure of contractual agreements that limit such access for investigators
Ancillary and post-trial care	30	Provisions, if any, for ancillary and post-trial care, and for compensation to those who suffer harm from trial participation
Dissemination policy	31a	Plans for investigators and sponsor to communicate trial results to participants, healthcare professionals, the public, and other relevant groups (eg, via publication, reporting in results databases, or other data sharing arrangements), including any publication restrictions
	31b	Authorship eligibility guidelines and any intended use of professional writers
	31c	Plans, if any, for granting public access to the full protocol, participant-level dataset, and statistical code

For peer review only

Appendices

Informed consent materials	32	Model consent form and other related documentation given to participants and authorised surrogates
Biological specimens	33	Plans for collection, laboratory evaluation, and storage of biological specimens for genetic or molecular analysis in the current trial and for future use in ancillary studies, if applicable

*It is strongly recommended that this checklist be read in conjunction with the SPIRIT 2013 Explanation & Elaboration for important clarification on the items. Amendments to the protocol should be tracked and dated. The SPIRIT checklist is copyrighted by the SPIRIT Group under the Creative Commons "Attribution-NonCommercial-NoDerivs 3.0 Unported" license.

For peer review only

BMJ Open

CASNET2: Evaluation of an Electronic Safety Netting cancer toolkit for the primary care electronic health record: protocol for a pragmatic stepped-wedge RCT

Journal:	*BMJ Open*
Manuscript ID	bmjopen-2020-038562.R1
Article Type:	Protocol
Date Submitted by the Author:	16-Jun-2020
Complete List of Authors:	Fleming, Susannah; University of Oxford, Nuffield Department of Primary Care Health Sciences<br>Nicholson, Brian; University of Oxford, Nuffield Dept Primary Care Health Sciences<br>Bhuiya, Afsana; North Central and East London Cancer Alliance<br>de Lusignan, Simon; University of Oxford, Nuffield Department of Primary Care Health Sciences; University of Surrey, Department of Clinical and Experimental Medicine<br>Hirst, Yasemin; University College London, Epidemiology and Public Health<br>Hobbs, Richard; University of Oxford, Nuffield Department of Primary Care Health Sciences<br>Perera, Rafael; University of Oxford, Primary Health Care<br>Sherlock, Julian; University of Oxford, Nuffield Department of Primary Care Health Sciences; University of Surrey, Department of Clinical and Experimental Medicine<br>Yonova , Ivelina; Royal College of General Practitioners, Research and Surveillance Centre<br>Bankhead, Clare; University of Oxford, Nuffield Department of Primary Care Health Sciences
**Primary Subject Heading**:	General practice / Family practice
Secondary Subject Heading:	Health informatics, Oncology
Keywords:	PRIMARY CARE, ONCOLOGY, Health informatics < BIOTECHNOLOGY & BIOINFORMATICS

SCHOLARONE™
Manuscripts

I, the Submitting Author has the right to grant and does grant on behalf of all authors of the Work (as defined in the below author licence), an exclusive licence and/or a non-exclusive licence for contributions from authors who are: i) UK Crown employees; ii) where BMJ has agreed a CC-BY licence shall apply, and/or iii) in accordance with the terms applicable for US Federal Government officers or employees acting as part of their official duties; on a worldwide, perpetual, irrevocable, royalty-free basis to BMJ Publishing Group Ltd ("BMJ") its licensees and where the relevant Journal is co-owned by BMJ to the co-owners of the Journal, to publish the Work in this journal and any other BMJ products and to exploit all rights, as set out in our licence.

The Submitting Author accepts and understands that any supply made under these terms is made by BMJ to the Submitting Author unless you are acting as an employee on behalf of your employer or a postgraduate student of an affiliated institution which is paying any applicable article publishing charge ("APC") for Open Access articles. Where the Submitting Author wishes to make the Work available on an Open Access basis (and intends to pay the relevant APC), the terms of reuse of such Open Access shall be governed by a Creative Commons licence – details of these licences and which Creative Commons licence will apply to this Work are set out in our licence referred to above.

Other than as permitted in any relevant BMJ Author's Self Archiving Policies, I confirm this Work has not been accepted for publication elsewhere, is not being considered for publication elsewhere and does not duplicate material already published. I confirm all authors consent to publication of this Work and authorise the granting of this licence.

For peer review only

CASNET2: Evaluation of an Electronic Safety Netting cancer toolkit for the primary care electronic health record: protocol for a pragmatic stepped-wedge RCT

For peer review only

Authors

Dr Susannah Fleming[1], Dr Brian D. Nicholson[1], Dr Afsana Bhuiya[2], Prof Simon de Lusignan[1,3,4], Dr Yasemin Hirst[5], Prof F.D. Richard Hobbs[1], Prof Rafael Perera[1], Julian Sherlock[1,3], Ivelina Yonova[1,3,4], Prof Clare R. Bankhead[1]

Affiliations

[1]*Nuffield Department of Primary Care Health Sciences, University of Oxford*
Susannah Fleming, Senior Quantitative Researcher, susannah.fleming@phc.ox.ac.uk
Brian D Nicholson, NIHR Academic Clinical Lecturer, brian.nicholson@phc.ox.ac.uk
F.D. Richard Hobbs, Nuffield Professor of Primary Care, richard.hobbs@phc.ox.ac.uk
Rafael Perera, Professor of Medical Statistics, rafael.perera@phc.ox.ac.uk
Clare R. Bankhead, Associate Professor, clare.bankhead@phc.ox.ac.uk

[1]*Nuffield Department of Primary Care Health Sciences, University of Oxford*
[3]*Department of Clinical and Experimental Medicine, University of Surrey, Guildford, GU2 7XH*
[4]*Royal College of General Practitioners Research and Surveillance Centre, Euston Square, London*
Simon de Lusignan, Professor of Primary Care and Clinical Informatics, simon.delusignan@phc.ox.ac.uk
Ivelina Yonova, Project Manager, ivelina.yonova@phc.ox.ac.uk

[1]*Nuffield Department of Primary Care Health Sciences, University of Oxford*
[3]*Department of Clinical and Experimental Medicine, University of Surrey, Guildford, GU2 7XH*
Julian Sherlock, SQL Developer, julian.sherlock@phc.ox.ac.uk

[2]*North Central and East London (NCEL) cancer alliance*
Afsana Bhuiya, Joint Chief Medical Officer, afsana.bhuiya1@nhs.net

[5]*Research Department of Behavioural Science and Health, University College London*
Yasemin Hirst, Research Associate, y.hirst@ucl.ac.uk

Corresponding Author

Brian D Nicholson, NIHR Academic Clinical Lecturer, brian.nicholson@phc.ox.ac.uk
Nuffield Department of Primary Care Health Sciences, University of Oxford

For peer review only - http://bmjopen.bmj.com/site/about/guidelines.xhtml

Word count: 3992 words

For peer review only

For peer review only - http://bmjopen.bmj.com/site/about/guidelines.xhtml

Abstract

Introduction

Safety-netting in primary care is best practice in cancer diagnosis, ensuring that patients are followed-up until symptoms are explained or have resolved. Currently, clinicians use haphazard manual solutions. The ubiquitous use of electronic health records (EHR) provides an opportunity to standardise safety-netting practices.

A new Electronic Safety-netting (E-SN) toolkit has been introduced to provide systematic ways to track and follow-up patients. We will evaluate the effectiveness of this toolkit, which is embedded in a major primary care clinical system in England (EMIS-Web).

Methods and analysis

We will conduct a stepped-wedge cluster RCT in 60 general practices within the RCGP Research and Surveillance Centre (RSC) network. Groups of 10 practices will be randomised into the active phase at 2-monthly intervals over 12 months. All practices will be activated for at least 2 months. The primary outcome is the primary care interval measured as days between the first recorded symptom of cancer (within the year prior to diagnosis) and the subsequent referral to secondary care. Other outcomes include referrals rates and rates of direct access cancer investigation.

Analysis of the clustered stepped-wedge design will model associations using a fixed effect for intervention condition of the cluster at each time step, a fixed effect for time and other covariates, and then include a random effect for practice and for patient to account for correlation between observations from the same centre and from the same participant.

Ethics and dissemination

Ethical approval has been obtained from the North West – Greater Manchester West NHS Research Ethics Committee (REC Reference 19/NW/0692). Results will be disseminated in peer-reviewed journals and conferences, and sent to participating practices. They will be published on the University of Oxford Nuffield Department of Primary Care and RCGP RSC websites.

Registration details

ISRCTN registry trial ID ISRCTN15913081.

Keywords

Primary Care, Cancer, Diagnostic Safety, Clinical Informatics, Randomised Controlled Trial.

Article Summary

- The safety-netting toolkit being evaluated is already implemented and integrated in the EMIS Web, a brand of electronic health record (EHR), but is rarely utilised

For peer review only - http://bmjopen.bmj.com/site/about/guidelines.xhtml

- Data collection will be carried out primarily through existing automated links between the practice EHR and the RCGP RSC, surveillance system, thereby limiting the additional research workload for participating practices
- We are reliant on the cancer diagnosis, generally made in specialist care, being recorded in the primary care EHR system in a consistent and timely manner.

Introduction

Background

Safety-netting is regarded as “best practice” in cancer diagnosis in primary care.(1) It aims to ensure patients do not drop through the healthcare net but are followed-up until symptoms are explained.(2) Our research highlights an absence of evidence on how best to safety-net, especially with patients with non-specific cancer symptoms.(1) Expert consensus, international survey data and interviews with GPs and patients show that effective patient communication, shared decision making and improved clinical systems are needed to ensure that tests and referrals are followed-up and recurrent consultations are identified in patients with unexplained symptoms.(3-5) To achieve this, significant improvements in electronic health record (EHR) utilisation, particularly data quality, are required, by integrating information and communication technology with clinical care.(6-8)

Whilst there are clear guidance and recommendations for safety netting in primary care,(9) successful implementation of these recommendations rely on resources available at general practices. Universally accessible NHS fail-safes do not exist to ensure tests are conducted, returned and reconciled.(10) Confusion exists about which staff member is responsible for test communication.(11) Patients can be unaware of their responsibility to follow up investigations and referrals, assuming “no news is good news,” and taking no action if they do not feel better or develop new symptoms.(12, 13) The success of a systems-based approach to safety-netting is jeopardised by inadequate administrative processes and marked variation in approaches to follow-up.(14) EHR based interventions show promise: trials in the United States (US) of electronic prompts increased the proportion of patients with cancer symptoms who receive follow-up.(8, 15-17) However, despite reporting enthusiasm for new initiatives, GPs do not always engage with new information technology, and this driven in part by social and technical factors, such as pop-up fatigue and information overload and being under-resourced (13, 14, 18-21).

An electronic safety-netting toolkit (E-SN toolkit) has been developed through consultation with GPs in the UK and was embedded within one of the major clinical systems in England - Egerton Medical Information System (EMIS) Web in May 2018. The toolkit is designed to replace existing verbal or paper methods of safety netting by providing effective means for tracking and follow-up by administrative staff. The E-SN toolkit proposes a rigorous, robust, traceable and auditable pro-active approach to tracking patients. It is designed to allow clinical data to be entered using templates, and diary entries to be generated (time reminders to check an action has been completed). Using diary entries, users can effectively follow up test requests, referrals, and non-specific but concerning symptoms. For instance, outstanding actions appear as “Alert Flags” to identify incomplete diary entries and can be collated. Although the E-SN toolkit is currently available to general practices using EMIS, practices have to proactively turn it on to implement it in their current practice.

This study aims to evaluate the effectiveness of an EHR safety-netting toolkit (E-SN toolkit) for use in primary care with patients with symptoms of cancer.

For peer review only - http://bmjopen.bmj.com/site/about/guidelines.xhtml

Study design

The study will employ a stepped wedge cluster-randomised controlled trial (SW-CRCT) randomising clusters, i.e. general practices, in blocks of 10 to the timing of activation of the E-SN toolkit. General practices will cross-over in these blocks to the activated phase every two months (Figure 1). The study will compare patient's primary care interval data for cancer diagnoses pre and post E-SN toolkit activation at all participating practices. The stepped-wedge design will ensure that time-related confounders such as seasonal variation should be accounted for.

This paper describes the protocol dated 7 October 2019 (version 1.5). Core trial information is given in Table 1.

The anticipated length of the study is 18 months. This consists of 3 months recruitment, followed by a 12 month period during which time the intervention will be introduced, and 3 months for analysis.

Methods and analyses

Outcomes and outcome definitions

Primary outcome: primary care interval for cancer diagnoses

The primary care interval is defined as the number of days between the first recorded symptoms of cancer (within the year prior to diagnosis) and subsequent referral for secondary cancer care. (22) In line with published research and guidelines on diagnostic intervals, we will search the patient record for coded symptoms during the year prior to diagnosis for all patients with a cancer diagnosis: one year is a trade-off between misattributing unrelated symptoms occurring more than a year before and missing symptoms of relevance by restricting to a shorter period.(22, 23).

Secondary Outcomes

Full details of the secondary outcomes are given in Table 2 and Figure 2.

Setting and participants

The study will be carried out in 60 English general practices that contribute data to the RCGP Research and Surveillance Centre (RSC) Network (24, 25) and use the EMIS Web EHR system. The RCGP RSC includes general practices in England. A full list of NHS Clinical Research Networks from which practices will be recruited may be found in Appendix A.

Inclusion and exclusion criteria

The inclusion criteria for general practices are as follows:

- Practice is actively contributing data to the RCGP RSC
- Utilises EMIS EHR system
- Data available for the previous 24 months

The exclusion criteria for general practices are:

- Practices that express an interest, but are not fully set up for data extraction
- Any practice already deploying the E-SN toolkit

For peer review only - http://bmjopen.bmj.com/site/about/guidelines.xhtml

All patients over the age of 18 at participating practices are eligible for inclusion in the study, unless they have opted out of data sharing.

Recruitment

The RCGP RSC network will identify potentially eligible practices and circulate the details of the study before and during the recruitment period. Practices will also be approached directly by the RCGP RSC Practice Liaison Officers (PLOs), who regularly visit practices to monitor their data quality and inform about current open studies. Expressions of Interest (EOI) will be obtained from all interested practices. An additional 12 practices will be identified to account for potential drop-outs during the study. All practices will be recruited at the start of the study prior to randomisation and implementation of the intervention.

General practices will receive reimbursement of up to £500 per practice for participation in the 12 month stepped-wedge intervention study.

Randomisation and blinding

The eligible practices will be ranked according to their list size from smallest to the largest by the RCGP RSC. These will then be stratified into 10 strata (by list size) such that each strata contains the same number of practices. This is based on the allocation of 10 practices per step. Practices will be randomised in blocks of 10 to the timing of activation of the E-SN toolkit and will cross-over in these blocks to the activated phase every two months. Therefore practices will contribute between 2 months and 12 months of E-SN toolkit-activated time (Figure 1).

A statistician (Rafael Perera, Nuffield Department of Primary Care Health Sciences, University of Oxford) who is independent to the intervention development and implementation will produce a stratified randomisation schedule so that within each strata, practices are randomly allocated to each of the 6 steps, with some replacement practices. The random sequence will be generated using R software. The allocation will be undertaken for all practices at the same time. Where replacement practices are required, these will be taken from the same strata where possible. If no replacement practices from the same strata are available a coin toss will determine whether the next highest or lowest strata will be used to provide replacement practices.

Given the practice change nature of the intervention, clinicians and practice managers will be aware when their practice has switched to the intervention period. Consulting patients providing outcome data will not be informed of the experimental nature of the E-SN Toolkit activation and therefore will be blind to the stage of study occurring in the practice they attended. Study personnel involved in extracting outcome data will be blind to the allocated order of the delivery of the intervention across the practices. All data management of extracted data to calculate the outcome measures will be conducted blinded to the timing of switching to intervention. Similar methods have been used in other implementation SW-CRTs.(26)

Intervention

The intervention consists of activation and the implementation of the E-SN toolkit at participating practices. All practices will receive training in the use of the E-SN toolkit from RCGP PSC Practice Liaison Officers prior to their switching date. Clinicians will be able to use the E-SN templates at their own

For peer review only - http://bmjopen.bmj.com/site/about/guidelines.xhtml

discretion during the active period, with no requirements on which patients should or should not receive safety netting. Clinical care of patients will continue as normal throughout the study.

The E-SN toolkit uses a templates to track cancer events like referrals, direct access tests and monitoring of low risk through read codes attached to diary entries. Expired diary entries would be identified using automated searches and actioned by the administration lead as appropriate. If an event was complete e.g. normal scan results done in 2 weeks and result back - then the diary entry is closed, resolving the episode. The E-SN toolkit has extra features such as popup alerts to remind any user there is an open diary entry and also allows the E-SN toolkit to popup automatically if a relevant code is used in the clinical records.

Further details and demonstration video are available from: https://www.uclh.nhs.uk/OurServices/ServiceA-Z/Cancer/NCV/MICa/Pages/Primarycareimprovement.aspx

Practices will all be encouraged to adhere to the schedule for switching and will receive part-payment for study initiation and then full payment when they adhere to the activation schedule.

Data extraction

Data extractions from all participating practices will correspond to two major time points: at the start of the introduction to the stepped wedge implementation of the E-SN toolkit, and at the end of the stepped wedge period (12 months later). At these two time points, consultation data from the participating practices will be obtained for the previous 24 months. Data from the previous 24 months is required to identify cancers detected in 12 months prior to start, plus the first recorded symptom of cancer during the primary care interval, allowing for this interval to cover up to a year prior to diagnosis.

Records from all patients who are adults aged 18 years and above, registered at participating practices and who have not opted out of sharing data for research purposes will be extracted. Pseudonymised patient information will include: demographic information of age, sex, dates of clinical consultations; coded consultation data for symptoms, diagnoses, tests ordered, and referrals made within the consultation records. Where a definitive diagnosis has been made (for cancer and non-cancer conditions), the clinical features recorded in the year prior to diagnosis will be captured. These actions will be achieved by extraction of the electronic health record rather than by hand searching notes.

Practices will be asked to search for open diary entries every week during the intervention period, and to download and save these to an Excel spreadsheet (locally). The central study research team will use these spreadsheets to track which diary entries are closed during the course of the intervention.

Data management

The principal data source for the this study are pseudonymised routinely collected care data extracted from general practices of the RCGP RSC network's database; there are over 500 general practices in this network. All general practices in the UK use an EHR to maintain patient medical records. Data are entered into a patient's computerised medical system as coded data or free text. The RCGP RSC extract only coded data, i.e. where the clinician codes a disease or symptom into their EHR system.(27)

The RCGP RSC has no role in updating clinical data recorded by clinicians as part of their consultation and care. However, the RCGP RSC practices do received focussed feedback about data quality around

For peer review only - http://bmjopen.bmj.com/site/about/guidelines.xhtml

its surveillance function, influenza other infections, vaccine update and effectiveness. This is done via team of PLOs and a dashboard.(28, 29)

RCGP RSC maintains an auditable trail for all the stages of data processing to ensure the quality of data are not compromised by the processing. For example, checking the prevalence of certain conditions and outliers revealed by the data is consistent with those reported in the literature. The standard operating procedures for data extraction and data processing, and data access are available from https://clininf.eu/index.php/information-governance/.

Discontinuations and withdrawals from the study

Each practice has the right to withdraw from the study at any time. Data from withdrawn practices will be included in analyses up to the point of withdrawal, unless they indicate that they wish to withdraw previously collected data from analysis.

Sample size

Practice lists sizes within the RCGP RSC are approximately 10,000. In England diagnosis rate of new cancer was 523/100,000 per year (2014/15).(30) Therefore we could expect 53 new cancers per year per practice. Therefore, in each 2 month step there would be 8 - 9 cancers per cluster.

The median primary care interval between first presentation and specialist referral(22, 31) is 5 days, interquartile range of 0-27.(32) Some cancers present with clear red flag symptoms leading to immediate specialist referral. Presentations of vague symptoms such as weight loss are less likely to be immediately referred and may benefit from using the safety-netting template. This symptom is associated with several cancers such as prostate, colorectal, lung, gastro-oesophageal, and pancreatic.(33) The median primary care interval for lung cancer is 14 days (interquartile range 2-45).(32) Using Stata 14 to conduct the sample size calculations we showed that with the design in Figure 1 and 60 practices we would be able to detect an effect size of 2 days with 80% power.

Currently approximately 19% of cancers are diagnosed following an emergency presentation.(34) With 9 cancers per step per cluster we would be powered to detect a difference of 5%.

Under another scenario of considering primary care intervals towards the 90th centile of 60 days, with 60 practices, entering the stepped wedge design in 6 steps we would be able to detect a minimal difference of 13 days. However, if we consider that these patients with longer delays are in the 90th centile, then instead of expected cancers per cluster per step of 9 – there would be around 1. This would allow us to detect a minimal difference of between 9 and 39 days dependent on the assumption of the distribution of the primary care delays.

Several scenarios are shown in Table 3, all based on 60 practices, entering in 6 blocks, with a 12 month pre-intervention period. In summary our main analysis will focus on all cancers, but we can conduct pre-specified subgroup analyses restricting to cancers that typically have longer delays.

Statistical analysis

In patients with a diagnosis of cancer we will calculate the primary outcome of primary care interval (time between first recorded symptom and referral), proportion diagnosed after emergency presentation and the recorded diagnosis. Amongst consulting patients we will calculate the rate of direct access cancer investigation and rates of referrals via 2 week wait, urgent and routine pathways, as well as the number of consultations during the primary care interval, and the template activation rate. A patient referred to: a 2 week wait pathway will be reviewed by a specialist within 2 weeks; an

For peer review only - http://bmjopen.bmj.com/site/about/guidelines.xhtml

urgent pathway will be reviewed as a priority but the exact timing varies between speciality and healthcare setting; a routine pathway requires specialist review but on a non-urgent basis.

Regarding the analysis of the stepped-wedge design and the effect of correlation of observations within clusters, will model the association using a fixed effect for the intervention condition of the cluster at each time step, a fixed effect for time and other covariates (e.g. changes in cancer guidelines), and then include a random effect for practice and a random effect for patient to account for correlation of the observations from the same centre and from the same participant.

Analyses will include all patients registered at participating practices. Practices that withdraw their agreement to participate will be included in analyses up to the point of withdrawal, unless they indicate that they wish to withdraw previously collected data from analysis.

Our primary analysis will be carried out on an intention to treat principle. Therefore, if any practice does not switch on the E-SN toolkit at the correct time for their cluster, we will carry out the analysis under the assumption that the E-SN toolkit was switched on at the correct time.

The primary care interval is defined from the presence of symptoms and referral in the primary care record. Symptoms of interest will include all symptoms included in the urgent referral guidelines including vague symptoms such as weight loss, tiredness, and back pain.(35, 36) Missing data for these variables in the record will be interpreted as the absence of symptoms or referral (respectively). Extracted data from the primary care record will be limited to the study period plus the preceding year, so symptoms recorded prior to this will not be visible to the research team.

The study will not have a formal data monitoring committee as patients will be receiving standard care through their GP, and all data will be extracted from routinely collected clinical notes.

Planned subgroups

Where applicable, subgroups will be:

- Patients in whom an E-SN toolkit entry was completed
- Patients diagnosed with cancer

Data display and reporting

We will combine or suppress any cells with small numbers (under 5) of observations to prevent any potential identification during the reporting of the results.

Patient and public involvement

We have recruited an experienced PPI representative to sit on the project steering committee. She has been involved in planning how PPI involvement will best fit in with the project, and will attend steering committee meetings to discuss project progress, as well as co-chairing workshops with patient representatives. We anticipate involving five additional patient representatives. The PPI collaborators will be involved in interpreting the findings of the study and identifying which of the pre-specified outcomes are of greatest importance to patients. This will allow them to consider whether there would be a beneficial effect in terms of patient perspectives, even if the E-SN Toolkit caused only a small change in outcomes.

For peer review only - http://bmjopen.bmj.com/site/about/guidelines.xhtml

Ethics and dissemination

Informed consent

Practices that are randomised to take part in the study will be provided with a welcome pack by their RCGP RSC PLO team. The pack includes a practice information leaflet (Appendix B) and practice poster (Appendix C), a copy of the protocol, and a copy of the ethical approval documentation, providing the practice with detailed information about the exact nature of the study; study requirements; the implications and constraints of the protocol; and any risks involved in taking part. Site agreements will be in place with each practice and it will be clearly stated that the practice is free to withdraw from the study at any time for any reason, without affecting their legal rights, and with no obligation to give the reason for withdrawal. Signing a site agreement will form consent for a practice to take part in the study.

No direct or active involvement will be required from consulting patients and we will not be seeking individual patient consent. The rationale for obtaining agreement at the cluster (practice) level is that the activation of the Toolkit will be through the EMIS Web software system and health care practitioners are the intended recipient of the intervention.(37, 38)

Patients who have opted out of sharing data are not processed by the RCGP RSC and therefore will not be accessed the research team and their data will not be extracted. RCGP RSC does keep a count of opt-out patients per practice as this is needed to interpret rates collected for surveillance, this runs at around 2% of the registered population.

A patient notification in the form of a poster will be displayed in all participating practices giving patients information about the study and how to opt out of data sharing. Outcome data will be extracted from coded data in the EHR by the SQL developer and provided in a pseudonymised form to the analysis team.

Safety reporting

As patients remain under their GP's care throughout the study, and serious adverse events such as death and hospitalisation unrelated to the study are expected in this patient group, no formal monitoring of serious adverse events will be carried out.

Quality assurance

The study may be monitored, or audited in accordance with the current approved protocol, GCP, relevant regulations and standard operating procedures by responsible individuals from the sponsor and the NHS trusts in which it is being carried out.

Ethical and regulatory considerations

Ethical approval has been obtained from the North West – Greater Manchester West NHS Research Ethics Committee (REC Reference 19/NW/0692). We have also obtained Health Research Authority (HRA) approval to carry out the study in the NHS. The study sponsor is the University of Oxford, UK (ctrg@admin.ox.ac.uk). All substantial amendments to the protocol will be submitted to the sponsor, the ethics committee, and the HRA, and, where necessary, their approval will be obtained.

The Investigator will ensure that this study is conducted in accordance with relevant regulations and with Good Clinical Practice. The University has a specialist insurance policy in place which would operate

For peer review only - http://bmjopen.bmj.com/site/about/guidelines.xhtml

in the event of any participant suffering harm as a result of their involvement in the research (Newline Underwriting Management Ltd, at Lloyd's of London). NHS indemnity operates in respect of the clinical treatment that is provided. All participants will continue to receive NHS care during and after the study.

Patient confidentiality

The study staff will ensure that the practices' patients' anonymity is maintained. The practice patients will be identified only by an ID number on all study documents and any electronic database.

All documents will be stored securely and only accessible by study staff and authorised personnel. The study will comply with the UK General Data Protection Regulation (GDPR) and Data Protection Act 2018, which require data to be anonymised as soon as it is practical to do so. Pseudonymisation will be carried out using the RCGP RSC standard processes and will ensure that it is not possible for research staff to link study data with data from other sources. Although multiple items will be extracted from individual clinical records, we have taken care to minimise the number of data items/variables made available for this analysis, for example using age rather than date of birth. RCGP RSC apply a process of statistical disclosure control (SDC) to ensure that individuals cannot be identified, even from aggregate data. For example, data might have to be exported by 5- or 10-year age-bands if there were small numbers in an individual year of birth.

Data are held on a protected by a firewall secure server at the University of Surrey, currently acting as the RCGP RSC's data and analysis hub. All in-bounded connections are blocked, but out-bounded connections are allowed upon approval by a senior staff member.

The secure server is managed by the Clinical Informatics and Health Outcomes Research Group, and will be moved to the University of Oxford where the Group is based. It meets the requirements of NHS Digital's Data Security and Privacy (DSP) toolkit.

Study management

The study will be overseen by three groups (see Table 4 for composition and roles of members.) Day-to-day management of the study will be primarily carried out by a core group of three researchers, meeting weekly to discuss any ongoing issues, with other members of the management group brought in to advise as necessary.

The study management group will meet quarterly and will provide pragmatic scientific and methodological support, management oversight, and will participate in dissemination activities and planning of future funding applications.

The study advisory board will meet quarterly and will focus on strategic oversight and progression reviews, and dissemination activities.

Dissemination

The primary results of the study will be published in a peer-reviewed journal publication. Additional dissemination may take place via peer-reviewed conference presentations, and additional journal publications. Participating general practices will receive a report of the main findings, and results will also be disseminated on both the University of Oxford Nuffield Department of Primary Care and RCGP RSC websites. Patients will not be directly informed of the results, but will be able to access results on the internet if they so wish.

Authorship of all publications will be determined in accordance with the International Committee of Medical Journal Editors (ICMJE) guidelines.

For peer review only - http://bmjopen.bmj.com/site/about/guidelines.xhtml

For peer review only

The final trial dataset will consist of a large quantity of pseudonymised participant-level data extracted from clinical records. Due to the potentially sensitive nature of this data, and to ensure compliance with relevant data protection law, this data will only be made accessible to the subgroup within the research team who will be carrying out the analysis, and there are no plans for granting public access to the data.

The full protocol will be made available as part of the study ISRCTN registration details following publication of this paper. Statistical code will be available from the authors on request.

Cancer diagnosis in the time of COVID-19

The COVID-19 pandemic has led to considerable changes in general practice, including a switch from predominantly face-to-face consultations to remote consultations by telephone or video call. In addition, many non-urgent hospital outpatient consultations and investigations have been cancelled or delayed, and it is likely that patients are less willing to consult with clinicians for what they perceive as "minor" conditions, due to a desire to reduce pressure on the health service. All of these factors are likely to impact on cancer diagnosis and referral.

As a result, the initiation of the CASNET2 study has been delayed in an attempt to minimise the effect of these factors on the results of the study, and to ensure that the results are generalizable. However, we also intend to carry out supplementary downloads from participating practices for the 12 months prior to March 2020 (baseline comparison period) and for the entire pandemic period, or until 31 October 2020, whichever is latest.

We will then conduct a before-and-after analysis of the following outcomes:

- Presenting symptoms of cancer
- Primary care interval for cancer diagnosis
- Proportion of cancers detected after emergency presentation
- Site and stage of new cancers
- Total time to diagnosis
- Number of consultations in primary care interval
- Rates of patients completing direct access cancer investigations
- Rates of patients referred by 2 week wait, urgent, and routine routes.

Author Statement

CRB, BDN, RH, RP, AB, YH, SdL, IY and JS contributed to writing the original grant application, on which the protocol was based. SF, CRB and BDN wrote the original protocol, with contributions from RP, AB, IY and SdL. SF, CRB, and BDN wrote the paper. All authors commented on the paper and approved the final version.

Funding

This work is supported by Cancer Research UK, Early Diagnosis Advisory Group (EDAG) grant number C48270/A27880. The CASNET2 COVID-19 extension (Cancer Diagnosis in the time of COVID-19) is funded by the University of Oxford Medical Sciences Division Covid-19 Research Response Fund (ref 0009070) Neither the funders nor the sponsor will have any role in the study design, data collection, management, analysis or interpretation of data, writing of reports, or the decision to submit reports for publication. CRB is supported by the NIHR Oxford Biomedical Research Centre and the Applied

For peer review only - http://bmjopen.bmj.com/site/about/guidelines.xhtml

Research Collaborative, Oxford. AB is funded by the NCEL Cancer Alliance. YH is funded by a Cancer Research UK Early Detection Grant (C38463/A26726). FDRH acknowledges his part-funding from the National Institute for Health Research (NIHR) School for Primary Care Research, the NIHR Collaboration for Leadership in Health Research and Care (CLARHC) Oxford, the NIHR Oxford Biomedical Research Centre (BRC), and the NIHR Oxford Medtech and In-Vitro Diagnostics Co-operative (MIC). SdeL has received commercial funding through his university for research funded by Seqirus, GSK, Lilly, Novo Nordisk, Astra Zenaca and Takeda principally for unrelated research in the areas of diabetes and vaccination. RP also received funding from the National Institute for Health Research (NIHR) Oxford Biomedical Research Centre, the NIHR Oxford Medtech and In-Vitro Diagnostics Co-operative, the NIHR Applied Research Collaboration Oxford and Thames Valley, and the Oxford Martin School.

Conflicts of interests

AB is the innovator of the E-SN toolkit and locally promotes the toolkit for the purposes of cancer care along its pathway. SF is a member of the National Body Temperature Measurement Group. SdeL is Director of the RCGP, as part of his academic work. CRB, YH, FDRH, BDN, RP, JS, and IY have no competing interests.

For peer review only

For peer review only - http://bmjopen.bmj.com/site/about/guidelines.xhtml

References

1. Nicholson BD, Mant D, Bankhead C. Can safety-netting improve cancer detection in patients with vague symptoms? BMJ. 2016;355:i5515.
2. Lyratzopoulos G, Vedsted P, Singh H. Understanding missed opportunities for more timely diagnosis of cancer in symptomatic patients after presentation. Br J Cancer. 2015;112 Suppl 1:S84-91.
3. Bankhead C, Heneghan C, Hewitson P, Thompson M. Safety netting to improve early cancer diagnosis in primary care: development of consensus guidelines. Department of Primary Health Care: University of Oxford; 2011.
4. Evans J, Ziebland S, MacArtney JI, Bankhead CR, Rose PW, Nicholson BD. GPs' understanding and practice of safety netting for potential cancer presentations: a qualitative study in primary care. Br J Gen Pract. 2018;68(672):e505-e11.
5. Nicholson BD, Goyder CR, Bankhead CR, Toftegaard BS, Rose PW, Thulesius H, et al. Responsibility for follow-up during the diagnostic process in primary care: a secondary analysis of International Cancer Benchmarking Partnership data. Br J Gen Pract. 2018;68(670):e323-e32.
6. Singh H, Spitzmueller C, Petersen NJ, Sawhney MK, Smith MW, Murphy DR, et al. Primary care practitioners' views on test result management in EHR-enabled health systems: a national survey. J Am Med Inform Assoc. 2013;20(4):727-35.
7. Callen JL, Westbrook JI, Georgiou A, Li J. Failure to follow-up test results for ambulatory patients: a systematic review. J Gen Intern Med. 2012;27(10):1334-48.
8. Improving diagnosis in health care [press release]. Washington, DC: The National Academies Press,2015.
9. NHS England Patient Safety Domain. Standards for the communication of patient diagnostic test results on

discharge from hospital. London: NHS England; 2016.
10. Litchfield I, Bentham L, Lilford R, McManus RJ, Hill A, Greenfield S. Test result communication in primary care: a survey of current practice. BMJ Qual Saf. 2015;24(11):691-9.
11. Litchfield IJ, Bentham LM, Lilford RJ, Greenfield SM. Test result communication in primary care: clinical and office staff perspectives. Fam Pract. 2014;31(5):592-7.
12. Singh H, Sittig DF. Were my diagnosis and treatment correct? No news is not necessarily good news. J Gen Intern Med. 2014;29(8):1087-9.
13. Litchfield IJ, Bentham LM, Lilford RJ, McManus RJ, Greenfield SM. Patient perspectives on test result communication in primary care: a qualitative study. Br J Gen Pract. 2015;65(632):e133-40.
14. Litchfield I, Bentham L, Hill A, McManus RJ, Lilford R, Greenfield S. Routine failures in the process for blood testing and the communication of results to patients in primary care in the UK: a qualitative exploration of patient and provider perspectives. BMJ Qual Saf. 2015;24(11):681-90.
15. Murphy DR, Laxmisan A, Reis BA, Thomas EJ, Esquivel A, Forjuoh SN, et al. Electronic health record-based triggers to detect potential delays in cancer diagnosis. BMJ Qual Saf. 2014;23(1):8-16.
16. Murphy DR, Thomas EJ, Meyer AN, Singh H. Development and Validation of Electronic Health Record-based Triggers to Detect Delays in Follow-up of Abnormal Lung Imaging Findings. Radiology. 2015;277(1):81-7.
17. Murphy DR, Wu L, Thomas EJ, Forjuoh SN, Meyer AN, Singh H. Electronic Trigger-Based Intervention to Reduce Delays in Diagnostic Evaluation for Cancer: A Cluster Randomized Controlled Trial. J Clin Oncol. 2015;33(31):3560-7.
18. Gross Z, Ozdilli J, Anastasiadis T, Ryan H. Using Cancer Decision Support Tools to support the early diagnosis of cancer 2017 [Available from: https://www.cancerresearchuk.org/sites/default/files/ace_cancer_decision_support_tools_final_report_v1.1_080517.pdf.
19. Legare F, Ratte S, Gravel K, Graham ID. Barriers and facilitators to implementing shared decision-making in clinical practice: update of a systematic review of health professionals' perceptions. Patient Educ Couns. 2008;73(3):526-35.

For peer review only - http://bmjopen.bmj.com/site/about/guidelines.xhtml

20. Usher-Smith J, Emery J, Hamilton W, Griffin SJ, Walter FM. Risk prediction tools for cancer in primary care. Br J Cancer. 2015;113(12):1645-50.
21. Murphy DR, Reis B, Kadiyala H, Hirani K, Sittig DF, Khan MM, et al. Electronic health record-based messages to primary care providers: valuable information or just noise? Arch Intern Med. 2012;172(3):283-5.
22. Weller D, Vedsted P, Rubin G, Walter FM, Emery J, Scott S, et al. The Aarhus statement: improving design and reporting of studies on early cancer diagnosis. Br J Cancer. 2012;106(7):1262-7.
23. Neal RD, Din NU, Hamilton W, Ukoumunne OC, Carter B, Stapley S, et al. Comparison of cancer diagnostic intervals before and after implementation of NICE guidelines: analysis of data from the UK General Practice Research Database. Br J Cancer. 2014;110(3):584-92.
24. Correa A, Hinton W, McGovern A, van Vlymen J, Yonova I, Jones S, et al. Royal College of General Practitioners Research and Surveillance Centre (RCGP RSC) sentinel network: a cohort profile. BMJ Open. 2016;6(4):e011092.
25. de Lusignan S, Correa A, Smith GE, Yonova I, Pebody R, Ferreira F, et al. RCGP Research and Surveillance Centre: 50 years' surveillance of influenza, infections, and respiratory conditions. Br J Gen Pract. 2017;67(663):440-1.
26. Kingsland M, Doherty E, Anderson AE, Crooks K, Tully B, Tremain D, et al. A practice change intervention to improve antenatal care addressing alcohol consumption by women during pregnancy: research protocol for a randomised stepped-wedge cluster trial. Implement Sci. 2018;13(1):112.
27. de Lusignan S. Codes, classifications, terminologies and nomenclatures: definition, development and application in practice. Inform Prim Care. 2005;13(1):65-70.
28. Liyanage H, Williams J, Byford R, Pathirannehelage S, de Lusignan S. Near-Real Time Monitoring of Vaccine Uptake of Pregnant Women in a Primary Care Sentinel Network: Ontological Case Definition Across Heterogeneous Data Sources. Stud Health Technol Inform. 2019;264:1855-6.
29. Pathirannehelage S, Kumarapeli P, Byford R, Yonova I, Ferreira F, de Lusignan S. Uptake of a Dashboard Designed to Give Realtime Feedback to a Sentinel Network About Key Data Required for Influenza Vaccine Effectiveness Studies. Stud Health Technol Inform. 2018;247:161-5.
30. Public Health England. Cancer Services: Demographics, Screening and diagnostics 2018 [Available from: https://fingertips.phe.org.uk/profile/cancerservices/data#page/0/.
31. Koo MM, Hamilton W, Walter FM, Rubin GP, Lyratzopoulos G. Symptom Signatures and Diagnostic Timeliness in Cancer Patients: A Review of Current Evidence. Neoplasia. 2018;20(2):165-74.
32. Swann R, McPhail S, Witt J, Shand B, Abel GA, Hiom S, et al. Diagnosing cancer in primary care: results from the National Cancer Diagnosis Audit. Br J Gen Pract. 2018;68(666):e63-e72.
33. Nicholson BD, Hamilton W, O'Sullivan J, Aveyard P, Hobbs FR. Weight loss as a predictor of cancer in primary care: a systematic review and meta-analysis. Br J Gen Pract. 2018;68(670):e311-e22.
34. Public Health England. National Cancer Registration and Analysis Service: Cancer Outcome Metrics 2018 2018 [Available from: http://www.ncin.org.uk/cancer_type_and_topic_specific_work/topic_specific_work/cancer_outcome_metrics.
35. NICE. Suspected cancer: recognition and referral (NG12). National Institute for Health and Care Excellence 2015.
36. Nicholson BD, Hamilton W, Koshiaris C, Oke JL, Hobbs FDR, Aveyard P. The association between unexpected weight loss and cancer diagnosis in primary care: a matched cohort analysis of 65,000 presentations. British Journal of Cancer. 2020;122(12):1848-56.
37. Dregan A, McDermott L, Ashworth M, McCann G, Charlton J, Yardley L, et al. Electronically-delivered, multi-component interventions to reduce unnecessary antibiotic prescribing in primary care. A cluster randomised trial using electronic health records (eCRT2). Health Technology Assessment. 2019;13/88/10.
38. Hutton JL. Are distinctive ethical principles required for cluster randomized controlled trials? Stat Med. 2001;20(3):473-88.

For peer review only - http://bmjopen.bmj.com/site/about/guidelines.xhtml

For peer review only

For peer review only

For peer review only - http://bmjopen.bmj.com/site/about/guidelines.xhtml

Figures and Tables

Figure 1. Stepped wedge design with 12 months pre-randomisation period. Pale blue cells represent inactive E-SN period, and purple cells represent active E-SN period.

Figure 2. Diagram showing the definitions of time-based outcomes

For peer review only

For peer review only - http://bmjopen.bmj.com/site/about/guidelines.xhtml

Table 1: WHO trial registration data set

Data category	Information
**Primary Registry and Trial Identifying Number**	ISRCTN: ISRCTN15913081
**Date of Registration in Primary Registry**	08/11/2019
**Secondary Identifying Numbers**	N/A
**Source(s) of Monetary or Material Support**	Cancer Research UK; Grant Codes: C48270/A27880
**Primary Sponsor**	University of Oxford
**Secondary Sponsor(s)**	None
**Contact for Public Queries**	susannah.fleming@phc.ox.ac.uk
**Contact for Scientific Queries**	clare.bankhead@phc.ox.ac.uk
**Public Title**	Testing an electronic safety netting system to help GPs follow up patients with worrying symptoms
**Scientific Title**	CASNET2: Evaluation of an e-safety netting cancer template in primary care: a pragmatic stepped-wedge RCT
**Countries of Recruitment**	United Kingdom
**Health Condition(s) or Problem(s) Studied**	E-safety netting (E-SN) toolkit
**Intervention(s)**	The researchers will recruit 60 general practices who are not currently using the E-SN toolkit, and randomise them in clusters (groups) of 10. Each cluster will have the E-SN toolkit turned on at a different time during the 12 months of the study. Once the E-SN toolkit is turned on, the GPs in the practice will be able to use it when caring for any patient they think would benefit from it, although it is expected that it will be of most use when treating patients with symptoms that might indicate cancer. The researchers will collect data from the electronic patient record system from the 12 months of the study and the 24 months before the start of the study to understand whether the introduction of the E-SN toolkit makes any difference to the diagnosis of cancer, and in particular to how quickly patients are diagnosed. The researchers will only extract records from patients who are over 18, and who have not opted out of the research.
**Key Inclusion and Exclusion Criteria**	GP practices will be eligible for inclusion under the following conditions:<br>1. They are actively contributing to the RCGP Research and Surveillance Centre database<br>2. They use the EMIS electronic health record system<br>3. They have data available for the previous 24 months<br>Within the participating practices, the researchers will seek to extract data from adult patients (aged over 18 years)<br><br>Exclusion criteria:<br>1. GP practices who are already using the E-SN toolkit will not be eligible for the study<br>2. The researchers will not extract data from any patient under 18, or from any patient who has opted out of data sharing for research purposes
**Study Type**	Other
**Date of First Enrollment**	25/11/2019
**Target Sample Size**	60
**Recruitment Status**	Not yet recruiting
**Primary Outcome(s)**	Primary care interval for cancer diagnoses measured as the time between the first recorded symptom of cancer and referral to secondary cancer care, during inactive and active E-SN phases
**Key Secondary Outcomes**	1. Proportion of cancers diagnosed after emergency presentation measured during inactive and active E-SN phases<br>2. Recorded new diagnoses in those who have a template activated, measured by cancer site and stage, and by non-cancer diagnosis, during the active E-SN stage<br>3. Total time to diagnosis measured from 1st recorded symptom to definitive diagnosis for all cancer diagnoses during the inactive and active E-SN phases and all diagnoses with template activation during the active E-SN phase<br>4. Number of GP consultations/patient between first record of symptom and cancer referral, measured during the inactive and active E-SN phase<br>5. Rates of patients completing direct access cancer investigations measured during the inactive and active E-SN phase<br>6. Rates of patients referred measured as 2-week wait, urgent, and routine, during the inactive and active E-SN phase<br>7. Timing of template activation within the primary care interval (from first symptom to referral) measured during the active E-SN phase<br>8. Template activation rate amongst consulting patients, both total and stratified by individual GP, measured during the active E-SN phase<br>9. The proportion of diary entries completed measured during the active E-SN phase

For peer review only - http://bmjopen.bmj.com/site/about/guidelines.xhtml

	10. The reason for template activation measured based on 20 high-level READ codes during the active E-SN phase<br>11. Symptoms leading to direct access to investigations measured during the active E-SN phase<br>12. Recorded vague symptoms in the template measured during the active E-SN phase<br>13. Demographic details of patients with activated templates measured during the active E-SN phase<br>14. GP type completing template (e.g. partner, locum, trainee) measured during the active E-SN phase<br>15. Diagnostic codes in patients with activated templates measured during the active E-SN phase

Table 2. Outcomes, measures, and time periods of measurement for primary and secondary outcomes

Outcome	Measure	Inactive Study Period	Active Study Period	Template activations only
**Primary outcome**				
**Primary care interval for cancer diagnoses**	Measured as the number of days between 1st recorded symptoms of cancer (within the year prior to diagnosis) and subsequent referral for secondary cancer care	X	X	
**Secondary outcomes**				
**Proportion of cancers diagnosed after emergency presentation**	Proportion of cancers for which diagnosis is made prior to referral, including following A&E or inpatient episodes. Where there is uncertainty regarding the route of diagnosis, the RCGP RSC network will contact the practice in an attempt to augment the data. Algorithms will also be developed to identify emergency presentations of cancer.	X	X	
**Recorded new diagnoses in those who have a template activated**	By cancer site and stage, and non-cancer diagnoses. Coded entries for all alternative diagnoses where the E-SN toolkit has been activated will be identified.		X	X
**Total time to diagnosis (from 1st recorded symptom to definitive diagnosis)**	Measured from 1st recorded symptom of cancer (within the previous year) to definitive diagnosis for all cancers diagnosed, and for all patients with an activated template	X	X	X (for non-cancer diagnoses)
**Number of GP consultations/patient between first record of symptom and cancer referral**	Measured as number of primary care consultations between the 1st recorded symptoms (within the year prior to diagnosis) and subsequent referral, per patient.	X	X	
**Rates of patients completing direct access cancer investigations**	Measured as the number of patients undergoing direct access cancer investigations (according to those specified in referral guidelines NG12) in each period divided by the person years of observation for that period.	X	X	
**Rates of patients referred for cancer**	Referrals rates via 2 week wait, urgent, and routine routes for all patients referred for specialist opinion to a secondary care cancer specialist	X	X	
**Timing of template activation**	Measured as the number of days between 1st recorded symptoms (within the year prior to diagnosis), and template activation and the number of days between template activation and subsequent referral.		X	X
**Template activation rate amongst consulting patients**	Measured as the number of patients with an activated template divided by the number of patients consulting, in each time period.<br>Both total rate and rate stratified by individual GP will be measured.		X	X
**Proportion of diary entries that were completed**	Measured as the number of diary entries that were completed divided by the number of diary entries that were opened.		X	X
**Reason for template activation**	The coded reasons for activating the template,<br>Based on 20 high level READ codes		X	X
**Symptoms leading to direct access to investigation**	All symptoms recorded in patients undergoing direct access cancer investigations	X	X	
**Recorded vague symptoms in the template**	All symptoms recorded within the template.		X	X
**Demographic details of patients with activated templates**	Age and sex of patients that had a template activated during the course of the trial.		X	X
**GP type completing templates**	Descriptive data on the type of GP that first activated the template (e.g. partner, locum, trainee)		X	X

For peer review only - http://bmjopen.bmj.com/site/about/guidelines.xhtml

**Diagnostic codes in patients with activated templates**	Diagnoses recorded after the activation of template.		X	X

Table 3. Scenarios for various assumptions used in the sample size calculations

Assumptions	All cancers (based on lung cancer)	All cancers	All cancers	All cancers	Restricting to only 90th centile delays	Restricting to only 90th centile delays
**Median (days)**	14	14	60	60	60	60
**Range (days)**	0-60	0 – 100	0 - 365	0 - 100	14 - 100	14 - 365
**N of cancers per step per cluster**	9	9	9	9	1	1
**Minimum detectable difference (days)**	2	5	13	4	9	39
**Notes**		Allowing greater range		Lower upper value for Primary Care Interval	Minimum set to median of all cancers	Min set to median of all cancers, but increased upper range

For peer review only

Table 4. Membership of groups overseeing the study

Member	Role	Day to day management	Management group	Advisory board
**Susannah Fleming**	Study co-ordinator	Yes	Yes	Yes
**Clare Bankhead**	PI	Yes	Yes	Yes
**Ivelina Yonova**	Project Manager	As necessary	Yes	Yes
**Brian Nicholson**	Co-PI	As necessary	Yes	Yes
**Simon de Lusignan**	Co-investigator		Yes	Yes
**Yasemin Hirst**	Co-investigator	As necessary	Yes	
**Afsana Bhuiya**	GP – Creator of E-SN toolkit	As necessary	Yes	
**Rafael Perera**	Statistician	As necessary	Yes	
**Julian Sherlock**	Programmer	As necessary	Yes	
**Sue Duncombe**	PPI representative	As necessary	Yes	Yes
**Jodie Moffatt**	Funder representative			Yes
**Rebecca Canning-Johns**	Independent statistician			Yes
**Richard Hobbs**	Co-investigator		Yes	
**Kathy Pritchard-Jones**	Advisor		Yes	

Appendices

Appendix A: List of study sites

Appendix B: Practice information sheet (RISP)

Appendix C: Patient poster

For peer review only - http://bmjopen.bmj.com/site/about/guidelines.xhtml

	Pre-randomisation period (months)						Post-randomisation cross-over period (months)					
Practices*	-12	-10	-8	-6	-4	-2	0-2	2-4	4-6	6-8	8-10	10-12
1-10												
11-20												
21-30												
31-40												
41-50												
51-60												

*Practices will be randomly allocated to the group and date of cross-over

For peer review only - http://bmjopen.bmj.com/site/about/guidelines.xhtml

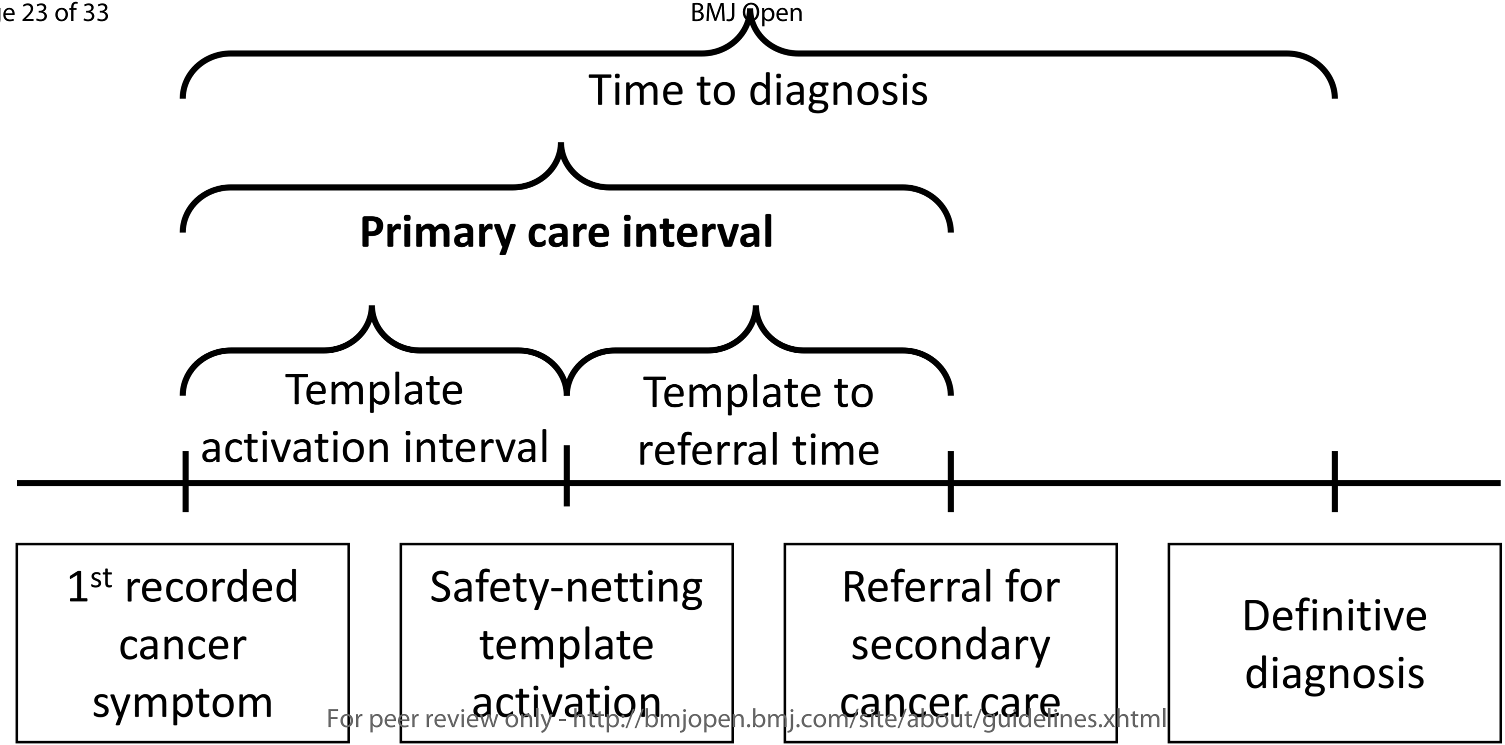

For peer review only - http://bmjopen.bmj.com/site/about/guidelines.xhtml

Appendix A: List of study sites

The list below is of NHS Local Clinical Research Networks within whose areas the general practices in the study will be located. At the time of publication, identification and recruitment of individual general practices for the study had not been completed. A complete list of individual sites will be available on request from the study team upon completion of study recruitment.

- North East and North Cumbria
- North West Coast
- Yorkshire and Humber
- Greater Manchester
- West Midlands
- West of England
- Thames Valley and South Midlands
- Eastern
- Kent, Surrey and Sussex
- Wessex
- South West Peninsula
- South London
- North West London

For peer review only

RISP
Research Information Sheet for Practices

**Study Title**	***CASNET2: Evaluation of an e-safety netting cancer template in primary care: a pragmatic stepped-wedge RCT***
**Principal Investigator(s) & Institution**	***Clare Bankhead, University of Oxford***
**NIHR Portfolio Ref**	**REC: TBC**<br>**IRAS ID: 269169**<br>**CPMS ID: TBC**
**Planned start date/ Duration of study recruitment at site**	Planned start date: 1 Sept 2019<br>12 months study duration – active period may be between 2 and 12 months depending on randomisation
**Study Funding Source**	Cancer Research UK
**Study Aim and Objectives**	The main aim of the study is to evaluate the E-safety netting (E-SN) toolkit, which is available as part of the EMIS electronic health record system. Our primary objective is to compare diagnostic outcomes for cancer (such as the time to diagnosis) during periods of time when the E-SN toolkit is turned off, compared with those during periods when the E-SN toolkit is turned on.<br>We also wish to compare consultation outcomes (such as the number of GP consultations before referral) during periods of time when the E-SN toolkit is turned off, compared with periods when the E-SN toolkit is turned on, to measure variation in the use of the E-SN toolkit between GP practices, and to describe the clinical situations for which the E-SN toolkit is used by GPs.<br>The E-SN toolkit is designed to assist with safety netting by providing a pro-active approach to tracking patients, ensuring that test requests, referrals and concerning symptoms are followed up in a timely manner.<br>We plan to recruit 60 general practices who are not currently using the E-SN toolkit, and randomise them in clusters (groups) of 10. Each cluster will have the E-SN toolkit turned on at a different time during the 12 months of the study. Once the E-SN toolkit is turned on, the GPs in the practice will be able to use it when treating any patient they think would benefit from it, although we expect that it will be of most useful when treating patients with symptoms that might indicate cancer.<br>We will collect data from the electronic patient record system from the 12 months of the study and the 24 months before the start of the study to understand whether the introduction of the E-SN toolkit makes any difference to the diagnosis of cancer, and particular to how quickly patients are diagnosed. We will only extract records from patients who are over 18, and who have not opted out of the research.

For peer review only - http://bmjopen.bmj.com/site/about/guidelines.xhtml

**Site target**	Not applicable as intervention is at practice rather than patient level
**Staff involvement at Practice**	General practitioners<br>Administrator
**Total funding paid**	Up to £500<br>• £100 for activating the template and attending the toolkit training provided by the study team<br>• £250 – for using the template as required during the study period<br>• £150 – to cover administrative time for keeping record of weekly searches and sending monthly reports to the study team
**Eligibility Criteria**	**Inclusion criteria:**<br>• Practice is actively contributing data to RCGP RSP<br>• Utilises EMIS electronic health records system<br>• Data available for the previous 24 months<br>• (Patients) Male or female, aged 18 years or above<br>**Exclusion criteria:**<br>• Practices that express an interest, but are not fully set up to start downloading data.<br>• Any practice already deploying the E-Safety netting toolkit within EMIS
**STUDY ACTIVITIES:**	
**Site Activities**	• Attend training in use of E-safety netting toolkit<br>• Activate E-safety netting toolkit in EMIS on date instructed by study team<br>• GPs to use E-safety netting toolkit in routine care when judged appropriate<br>• Administrator to perform weekly search in EMIS for open diary entries and download these to an Excel spreadsheet<br>• Downloaded Excel spreadsheets of open diary entries to be provided on a monthly basis to study team.
**Patient Activities**	None
**Recruitment data process**	Recruitment will be reported by the study team, so no recruitment upload is required by practices.
**Study specific training**	• GPs at participating sites will receive training in use of the E-safety netting toolkit.<br>• The study team will also provide training and support for administrators to assist with weekly searching for open diary entries.
**Possible risks**	• Use of the E-safety netting toolkit may cause a slight increase in overall consultation length, although this is expected to be of the order of tens of seconds<br>• Reduced trust in the health system due to patients becoming aware that their data is being used for research without explicit consent
**GDPR and confidentiality information**	Responsible members of the University of Oxford may be given access to data for monitoring and/or audit of the study to ensure that the research is complying with applicable regulations.<br>Data protection regulation requires that we state the legal basis for processing information about your practice and the patients registered there. In the case of research, this is 'a task in the public interest.' The University of Oxford is the data controller and is responsible for looking after this information and using it properly.<br>We will be using information from medical records in order to undertake this study. Patients who have opted not to share their information for disease surveillance will be respected by RCGP RSC and by the research team. The research team will not extract records of patients who have registered opt-out codes in the GP information system.

For peer review only - http://bmjopen.bmj.com/site/about/guidelines.xhtml

	Outcome data will be extracted from the EHR by the SQL developer and provided in a pseudonymised form to the analysis team.<br><br>You can find out more about how we use information from your practice and the patients registered there by contacting clare.bankhead@phc.ox.ac.uk or susannah.fleming@phc.ox.ac.uk
**Withdrawal from study**	Participation in the study is voluntary. If a practice chooses to withdraw from the study, data collected up to that point will be included in analyses. A practice may request upon withdrawal that data collected up to the point of withdrawal should not be used for analysis.<br>Practices who withdraw will receive the payment for activation and training, but payments for using the template and sending monthly reports of searches will be provided pro-rata for the period of time spent with the E-SN toolkit turned on before withdrawal.
**Making a complaint**	The University of Oxford, as Sponsor, has appropriate insurance in place in the unlikely event that you suffer any harm as a direct consequence of your participation in this study. NHS indemnity operates in respect of the clinical treatment which is provided.<br>If you wish to complain about any aspect of the way in which you have been approached or treated, or how your information is handled during the course of this study, you should contact Clare Bankhead on 01865 289351, or by email: clare.bankhead@phc.ox.ac.uk. Alternatively, you may contact the University of Oxford Clinical Trials and Research Governance (CTRG) office on 01865 616480, or the head of CTRG, email ctrg@admin.ox.ac.uk.
**Research Sponsor**	University of Oxford<br>ctrg@admin.ox.ac.uk
**General study contact**	Manasa Tripathy, Practice Liason Officer<br>manasa.tripathy@phc.ox.ac.uk

For peer review only - http://bmjopen.bmj.com/site/about/guidelines.xhtml

CASNET2 Study

RCGP
Research & Surveillance Centre Practice

This practice is taking part in the CASNET2 study. This is a study run by researchers at the University of Oxford looking at a new electronic safety netting tool which is part of the practice computer system. The tool helps GPs to make sure that patients get followed up if they have symptoms that might lead to a new diagnosis.

The researchers are hoping to find out whether the tool helps to reduce the time it takes for people to get a diagnosis. They will be using anonymised patient data from the practice computer system to find out how well the tool works. Using data in this way will not affect your privacy or care, and no-one will be able to identify you personally, but please speak to your GP if you have any concerns, or if you wish to opt out of the study.

For further information please visit http://www.rcgp.org.uk/rsc or contact:

Prof Simon de Lusignan
RSC Director
MedicalDirectorRSC@rcgp.org.uk

Dr Lucy Moore
Research Facilitator
practiceenquiries@phc.ox.ac.uk

Royal College of General Practitioners (RCGP) Research and Surveillance Centre (RSC)

For peer review only - http://bmjopen.bmj.com/site/about/guidelines.xhtml

SPIRIT

STANDARD PROTOCOL ITEMS: RECOMMENDATIONS FOR INTERVENTIONAL TRIALS

SPIRIT 2013 Checklist: Recommended items to address in a clinical trial protocol and related documents*

Section/item	ItemNo	Description	Page(s)
**Administrative information**			
Title	1	Descriptive title identifying the study design, population, interventions, and, if applicable, trial acronym	Page 1
Trial registration	2a	Trial identifier and registry name. If not yet registered, name of intended registry	Page 2
	2b	All items from the World Health Organization Trial Registration Data Set	Table 1
Protocol version	3	Date and version identifier	Page 4
Funding	4	Sources and types of financial, material, and other support	Page 12
Roles and responsibilities	5a	Names, affiliations, and roles of protocol contributors	Pages 1 and 12
	5b	Name and contact information for the trial sponsor	Page 9
	5c	Role of study sponsor and funders, if any, in study design; collection, management, analysis, and interpretation of data; writing of the report; and the decision to submit the report for publication, including whether they will have ultimate authority over any of these activities	Page 12
	5d	Composition, roles, and responsibilities of the coordinating centre, steering committee, endpoint adjudication committee, data management team, and other individuals or groups overseeing the trial, if applicable (see Item 21a for data monitoring committee)	Page 10
**Introduction**			

For peer review only

Background and rationale	6a	Description of research question and justification for undertaking the trial, including summary of relevant studies (published and unpublished) examining benefits and harms for each intervention	Page 3
	6b	Explanation for choice of comparators	Pages 3-4
Objectives	7	Specific objectives or hypotheses	Page 3
Trial design	8	Description of trial design including type of trial (eg, parallel group, crossover, factorial, single group), allocation ratio, and framework (eg, superiority, equivalence, noninferiority, exploratory)	Pages 3-4

Methods: Participants, interventions, and outcomes

Study setting	9	Description of study settings (eg, community clinic, academic hospital) and list of countries where data will be collected. Reference to where list of study sites can be obtained	Page 4 and Appendix A
Eligibility criteria	10	Inclusion and exclusion criteria for participants. If applicable, eligibility criteria for study centres and individuals who will perform the interventions (eg, surgeons, psychotherapists)	Page 4
Interventions	11a	Interventions for each group with sufficient detail to allow replication, including how and when they will be administered	Pages 5-6
	11b	Criteria for discontinuing or modifying allocated interventions for a given trial participant (eg, drug dose change in response to harms, participant request, or improving/worsening disease)	n/a
	11c	Strategies to improve adherence to intervention protocols, and any procedures for monitoring adherence (eg, drug tablet return, laboratory tests)	Page 6
	11d	Relevant concomitant care and interventions that are permitted or prohibited during the trial	Page 5

For peer review only - http://bmjopen.bmj.com/site/about/guidelines.xhtml

Outcomes	12	Primary, secondary, and other outcomes, including the specific measurement variable (eg, systolic blood pressure), analysis metric (eg, change from baseline, final value, time to event), method of aggregation (eg, median, proportion), and time point for each outcome. Explanation of the clinical relevance of chosen efficacy and harm outcomes is strongly recommended	Page 4 and Table 2
Participant timeline	13	Time schedule of enrolment, interventions (including any run-ins and washouts), assessments, and visits for participants. A schematic diagram is highly recommended (see Figure)	Figure 1
Sample size	14	Estimated number of participants needed to achieve study objectives and how it was determined, including clinical and statistical assumptions supporting any sample size calculations	Page 7
Recruitment	15	Strategies for achieving adequate participant enrolment to reach target sample size	Pages 4-5
**Methods: Assignment of interventions (for controlled trials)**			
Allocation:			
Sequence generation	16a	Method of generating the allocation sequence (eg, computer-generated random numbers), and list of any factors for stratification. To reduce predictability of a random sequence, details of any planned restriction (eg, blocking) should be provided in a separate document that is unavailable to those who enrol participants or assign interventions	Page 5
Allocation concealment mechanism	16b	Mechanism of implementing the allocation sequence (eg, central telephone; sequentially numbered, opaque, sealed envelopes), describing any steps to conceal the sequence until interventions are assigned	Page 5
Implementation	16c	Who will generate the allocation sequence, who will enrol participants, and who will assign participants to interventions	Page 5
Blinding (masking)	17a	Who will be blinded after assignment to interventions (eg, trial participants, care providers, outcome assessors, data analysts), and how	Page 5

For peer review only - http://bmjopen.bmj.com/site/about/guidelines.xhtml

	17b	If blinded, circumstances under which unblinding is permissible, and procedure for revealing a participant's allocated intervention during the trial	n/a
**Methods: Data collection, management, and analysis**			
Data collection methods	18a	Plans for assessment and collection of outcome, baseline, and other trial data, including any related processes to promote data quality (eg, duplicate measurements, training of assessors) and a description of study instruments (eg, questionnaires, laboratory tests) along with their reliability and validity, if known. Reference to where data collection forms can be found, if not in the protocol	Page 6
	18b	Plans to promote participant retention and complete follow-up, including list of any outcome data to be collected for participants who discontinue or deviate from intervention protocols	Page 6
Data management	19	Plans for data entry, coding, security, and storage, including any related processes to promote data quality (eg, double data entry; range checks for data values). Reference to where details of data management procedures can be found, if not in the protocol	Page 6
Statistical methods	20a	Statistical methods for analysing primary and secondary outcomes. Reference to where other details of the statistical analysis plan can be found, if not in the protocol	Page 7-8
	20b	Methods for any additional analyses (eg, subgroup and adjusted analyses)	Page 8
	20c	Definition of analysis population relating to protocol non-adherence (eg, as randomised analysis), and any statistical methods to handle missing data (eg, multiple imputation)	Page 7
**Methods: Monitoring**			

Data monitoring	21a	Composition of data monitoring committee (DMC); summary of its role and reporting structure; statement of whether it is independent from the sponsor and competing interests; and reference to where further details about its charter can be found, if not in the protocol. Alternatively, an explanation of why a DMC is not needed	Page 8
	21b	Description of any interim analyses and stopping guidelines, including who will have access to these interim results and make the final decision to terminate the trial	n/a
Harms	22	Plans for collecting, assessing, reporting, and managing solicited and spontaneously reported adverse events and other unintended effects of trial interventions or trial conduct	Page 9
Auditing	23	Frequency and procedures for auditing trial conduct, if any, and whether the process will be independent from investigators and the sponsor	Page 9
**Ethics and dissemination**			
Research ethics approval	24	Plans for seeking research ethics committee/institutional review board (REC/IRB) approval	Page 9
Protocol amendments	25	Plans for communicating important protocol modifications (eg, changes to eligibility criteria, outcomes, analyses) to relevant parties (eg, investigators, REC/IRBs, trial participants, trial registries, journals, regulators)	Page 9
Consent or assent	26a	Who will obtain informed consent or assent from potential trial participants or authorised surrogates, and how (see Item 32)	Page 8
	26b	Additional consent provisions for collection and use of participant data and biological specimens in ancillary studies, if applicable	Pages 8-9
Confidentiality	27	How personal information about potential and enrolled participants will be collected, shared, and maintained in order to protect confidentiality before, during, and after the trial	Page 9
Declaration of interests	28	Financial and other competing interests for principal investigators for the overall trial and each study site	Page 12

For peer review only

Access to data	29	Statement of who will have access to the final trial dataset, and disclosure of contractual agreements that limit such access for investigators	Page 10
Ancillary and post-trial care	30	Provisions, if any, for ancillary and post-trial care, and for compensation to those who suffer harm from trial participation	Page 9
Dissemination policy	31a	Plans for investigators and sponsor to communicate trial results to participants, healthcare professionals, the public, and other relevant groups (eg, via publication, reporting in results databases, or other data sharing arrangements), including any publication restrictions	Page 10
	31b	Authorship eligibility guidelines and any intended use of professional writers	Page 10
	31c	Plans, if any, for granting public access to the full protocol, participant-level dataset, and statistical code	Page 10
**Appendices**			
Informed consent materials	32	Model consent form and other related documentation given to participants and authorised surrogates	Appendices B and C
Biological specimens	33	Plans for collection, laboratory evaluation, and storage of biological specimens for genetic or molecular analysis in the current trial and for future use in ancillary studies, if applicable	n/a

*It is strongly recommended that this checklist be read in conjunction with the SPIRIT 2013 Explanation & Elaboration for important clarification on the items. Amendments to the protocol should be tracked and dated. The SPIRIT checklist is copyrighted by the SPIRIT Group under the Creative Commons "Attribution-NonCommercial-NoDerivs 3.0 Unported" license.

For peer review only
